# Pan-European study of genotypes and phenotypes in the Arabidopsis relative *Cardamine hirsuta* reveals how adaptation, demography, and development shape diversity patterns

**DOI:** 10.1371/journal.pbio.3002191

**Published:** 2023-07-18

**Authors:** Lukas Baumgarten, Bjorn Pieper, Baoxing Song, Sébastien Mane, Janne Lempe, Jonathan Lamb, Elizabeth L. Cooke, Rachita Srivastava, Stefan Strütt, Danijela Žanko, Pedro GP Casimiro, Asis Hallab, Maria Cartolano, Alexander D. Tattersall, Bruno Huettel, Dmitry A. Filatov, Pavlos Pavlidis, Barbara Neuffer, Christos Bazakos, Hanno Schaefer, Richard Mott, Xiangchao Gan, Carlos Alonso-Blanco, Stefan Laurent, Miltos Tsiantis

**Affiliations:** 1 Department of Comparative Development and Genetics, Max Planck Institute for Plant Breeding Research, Cologne, Germany; 2 Department of Biology, University of Oxford, Oxford, United Kingdom; 3 Jardim Botânico do Faial, Azores, Portugal; 4 Max Planck Genome Centre Cologne, Max Planck Institute for Plant Breeding Research, Cologne, Germany; 5 Institute of Computer Science, Foundation for Research and Technology, Crete, Greece; 6 Department of Botany, University of Osnabrück, Osnabrück, Germany; 7 Department Life Science Systems, School of Life Sciences, Technical University of Munich, Freising, Germany; 8 Department of Genetics, Evolution and Environment, University College London, London, United Kingdom; 9 Department of Plant Molecular Genetics, Centro Nacional de Biotecnología (CNB), Consejo Superior de Investigaciones Científicas (CSIC), Madrid, Spain; Institute of Science and Technology Austria (IST Austria), AUSTRIA

## Abstract

We study natural DNA polymorphisms and associated phenotypes in the Arabidopsis relative *Cardamine hirsuta*. We observed strong genetic differentiation among several ancestry groups and broader distribution of Iberian relict strains in European *C*. *hirsuta* compared to Arabidopsis. We found synchronization between vegetative and reproductive development and a pervasive role for heterochronic pathways in shaping *C*. *hirsuta* natural variation. A single, fast-cycling *ChFRIGIDA* allele evolved adaptively allowing range expansion from glacial refugia, unlike Arabidopsis where multiple *FRIGIDA* haplotypes were involved. The Azores islands, where Arabidopsis is scarce, are a hotspot for *C*. *hirsuta* diversity. We identified a quantitative trait locus (QTL) in the heterochronic SPL9 transcription factor as a determinant of an Azorean morphotype. This QTL shows evidence for positive selection, and its distribution mirrors a climate gradient that broadly shaped the Azorean flora. Overall, we establish a framework to explore how the interplay of adaptation, demography, and development shaped diversity patterns of 2 related plant species.

## Abstract 2

This study explores how the interplay of adaptation, demography and development shaped diversity patterns in Cardamine hirsuta, a close relative of Arabidopsis, and shows that a QTL in the heterochronic SPL9 gene underlies a distinctive morphotype discovered in the Azores islands.

## Introduction

Comparative analysis of related taxa offers the opportunity to investigate how the interplay of adaptation, historical contingency (the outcome of past events that are often random), and developmental constraints shaped phenotypic and genetic diversity [1–4]. In doing so, it also helps understand the repeatability of evolution and how balance between conservation and divergence in different molecular pathways shapes trait diversity [5]. Over the past 4 decades, studies of the model organism *Arabidopsis thaliana* have yielded fundamental insights into how genotypes are translated into phenotypes during development of seed plants [6,7]. Natural variation studies have uncovered causal variants that underlie ecologically important trait diversity in *A*. *thaliana* [8–15]. At the same time, studies of naturally occurring polymorphisms revealed *A*. *thaliana’s* demographic history and population structure and allowed investigations of the effects of demography on trait diversity [16–19]. *Cardamine hirsuta*, a close relative of *A*. *thaliana*, offers excellent opportunities to understand diversity in a comparative fashion because it shares many attributes that make a good model organism, while also showing differences in key traits including floral morphology, seed dispersal, and leaf shape [20]. These attributes have already been used to identify genes and processes underlying differences between the 2 species. These include the subdivision of the *C*. *hirsuta* leaf into distinct leaflets versus the simple leaf of *A*. *thaliana* and the explosive seed dispersal of *C*. *hirsuta* versus the passive dispersal in *A*. *thaliana* [21–24].

*C*. *hirsuta* also shows natural variation for many traits, which provides an untapped resource for comparative studies of diversity at the population level [25]. A major focus of natural variation studies in *A*. *thaliana* has been flowering time, a key life history trait that marks the onset of reproduction and contributes to local adaptation [26–29]. Overall, these Arabidopsis studies indicate that natural allelic variation in flowering time genes often affects physiological and morphological traits pleiotropically and can also influence for example seed germination and water use efficiency [30,31]. Such combined effects of flowering time loci may allow emergence of integrated life history strategies that support ecological adaptations [32–36]. A quantitative trait locus (QTL) cloned in *C*. *hirsuta* provided evidence for trait integration between flowering time and complex leaf morphology [25]. Specifically, a weak allele of the floral repressor *FLOWERING LOCUS C* (*FLC*) acts heterochronically by accelerating the rate of transition from simpler juvenile leaves to adult leaf forms bearing more leaflets. This synchronization of flowering time and leaf development resulted in more leaflets being produced in anticipation of reproduction, which may support resource allocation to the next generation in the form of seeds [25]. Thus, genetic variation at age-dependent pathways likely contributed to natural variation for shoot morphology in *C*. *hirsuta*. Notably, many traits are under age-dependent control in seed plants, including stress responses and nutritional status [37–40], thus potentially allowing their integrated developmental control in diverse natural settings [41,42]. However, the degree to which heterochronic pathways broadly shape species-wide morphological variation in *C*. *hirsuta*, and the ecological factors driving its maintenance remain unknown. Addressing this question and studying the degree of conservation versus divergence of pathways shaping natural trait variation in *C*. *hirsuta* and *A*. *thaliana* requires a comprehensive survey of genetic and phenotypic diversity in *C*. *hirsuta*.

Here, we use genome-wide single nucleotide polymorphism (SNP) data to analyze the demographic history of European and Macaronesian populations of *C*. *hirsuta*. We find that *C*. *hirsuta* from the Iberian Peninsula and the Macaronesian Islands retain considerable genetic diversity, thus resembling relict populations of *A*. *thaliana*. However, *C*. *hirsuta* relict-like strains extend more than *A*. *thaliana* relicts into mainland Europe and therefore have been more able to establish outside glacial refugia. We then use our strain panel to study genetic control of leaflet number and flowering time. We find evidence for their correlated control by the flowering time loci *FRIGIDA* and *FLC* as well as a component of the trehalose biosynthesis pathway. Furthermore, polymorphisms at *FRIGIDA* display a distinct signature of positive selection, providing a striking example of parallel adaptive evolution with *A*. *thaliana*, where multiple loss-of-function alleles drive flowering time adaptation in northern latitudes [43]. However, in contrast to *A*. *thaliana*, a single loss-of-function haplotype dominates variation in *C*. *hirsuta*, highlighting how the balance between stochastic and deterministic forces drove parallel trait evolution in these 2 species. Finally, we find abundant diverse *C*. *hirsuta* populations in the Azorean islands where seasonality is reduced compared to continental Europe. In exploring the genetic basis for the Azores colonization, we found a QTL cluster that affects age-dependent variation in leaf form without pronounced effect on flowering time and includes a derived allele of the heterochronically acting transcription factor SQUAMOSA PROMOTER BINDING-LIKE 9 (SPL9), which we validate in transgenic assays. This allele shows a polarized East–West geographic distribution in the Azores that correlates with environmental differences, particularly water availability. The same genomic region shows evidence for positive selection at and around the *SPL9* locus, indicating that modulation of *SPL9*-dependent variation in shoot development contributed to adaptation of *C*. *hirsuta* in its ecological niche of the Western Azores where plants experience year-round conditions permissive for growth, punctuated by dry summer spells. Our study establishes *C*. *hirsuta* as a valuable model for comparative studies at the population level in plants and shows that heterochronic pathways had a major contribution to natural variation in this species under diverse ecological conditions.

## Results

### Deep genetic structure and a recent range expansion characterize genetic diversity in *C*. *hirsuta*

We set out to understand the demographic history of *C*. *hirsuta* and compare it with *A*. *thaliana*. Given that the 2 species share similar life cycles, we reasoned that if their distribution and colonization history was shaped by similar ecological forces, then this similarity might be preserved in common patterns of extant genetic diversity in the 2 taxa. Conversely, large differences in the evolutionary trajectories of the 2 species might have left imprints in the distribution of genetic diversity patterns.

**Fig 1:**
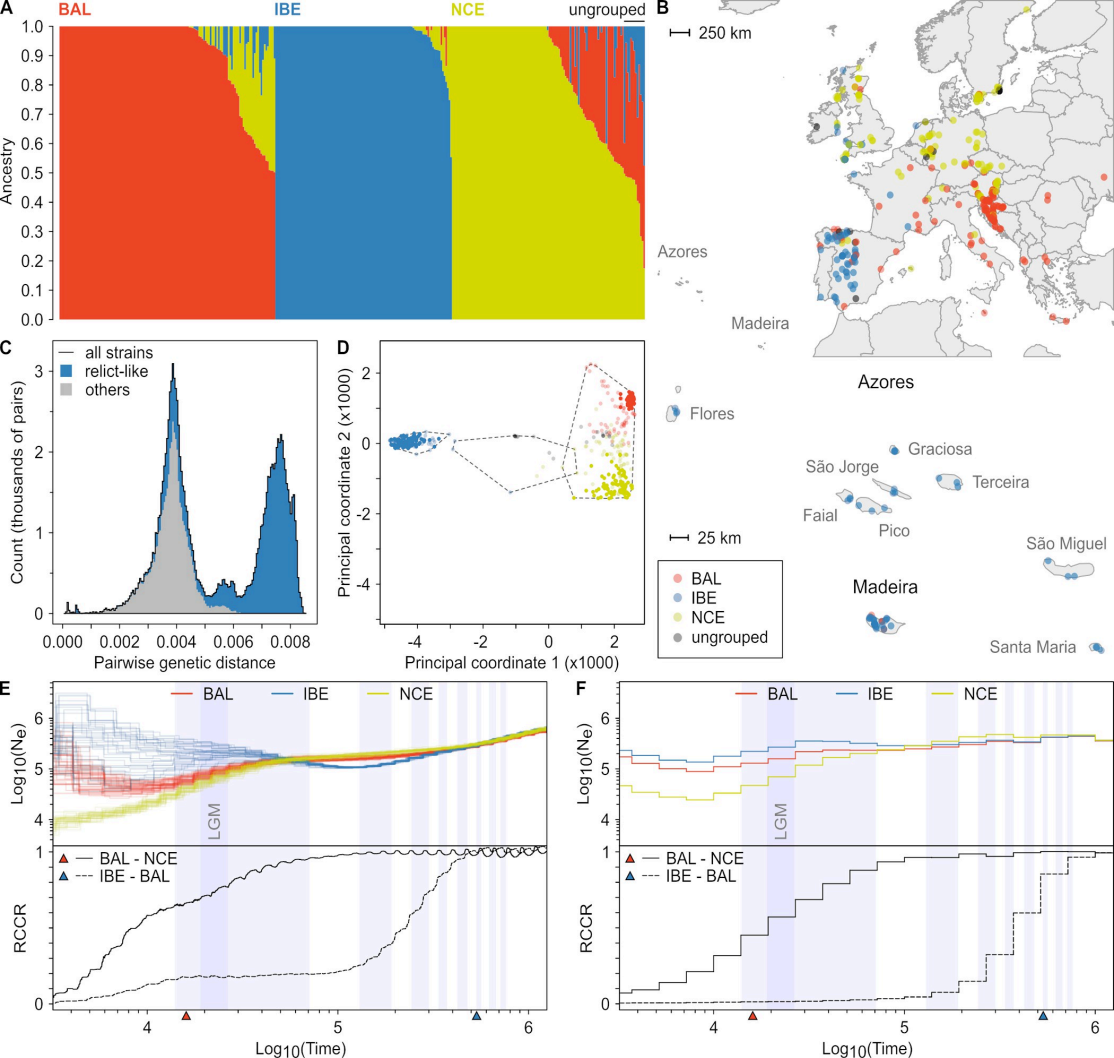
Population structure and demography of *Cardamine hirsuta*. (**A**) *ADMIXTURE* analysis of *C*. *hirsuta* strains after filtering for close relatedness (*n =* 358) reveals 3 major ancestry groups. The number of clusters that best fitted the data was found to be 3 (see also in S1A Fig). Each vertical bar represents a strain, where the colors indicate admixture proportions for the 3 ancestry groups. Strains were assigned to the ancestry group for which the proportion of ancestry was at least 0.5. The ancestry groups were named according to the main sampling location of their respective strains: BAL–Balkan; IBE–Iberia; NCE–Northern Central Europe. Strains with proportions less than 0.5 for all ancestry groups were categorized as ungrouped (top right). (**B**) The geographical distribution of the *C*. *hirsuta* ancestry groups in Western Europe. Each point represents the collection site of a strain and is colored according to the ancestry group it belonged to, with ungrouped strains shown in gray. The Macaronesian islands of the Azores and Madeira are shown at a smaller scale below the map. Map layers were made with Natural Earth and [142]. (**C**) The distribution of pairwise genetic distances (PGDs) indicates a deep split between groups of *C*. *hirsuta* strains. A histogram is shown of PGD between all possible pairs of strains in which the numbers of pairs in each bin are plotted against the PGD. The black outline shows the PGD of all strains in our sample. The presence of 2 major modes in the distribution, of which one at high genetic distance, indicated a group of strains in our sample that is highly differentiated from the others. Hierarchical clustering revealed a group of relict-like strains that was responsible for the second major mode in the distribution. PGDs including 1 or 2 relict-like strains are shown in blue, and PGDs not including those are shown in gray. (**D**) Identification of groups of *C*. *hirsuta* strains that are highly differentiated from each other based on multidimensional scaling and hierarchical clustering of the PGD. The first 2 PCs are plotted against each other where each point is a strain, colored according to the *ADMIXTURE* ancestry group it belonged to, with ungrouped strains shown in dark gray. Strains with ancestry in only a single ancestry group in the *ADMIXTURE* analysis are shown by darker shades versus lighter shades for admixed strains. Hierarchical clustering of the PGD matrix revealed that the separation of the strains along PC1 represented the 3 distinct groups of strains shown enclosed by dashed lines. The groups on the left and in the middle were responsible for the second major mode in the distribution of PGD (Figs 1C, S1B and S1C). Those 2 groups are shown here in blue and gray. (**E**, **F**) Piecewise constant effective population sizes (N_e_) as a function of time for the 3 ancestry groups using *MSMC2* (**E**) and *relate* (**F**), and estimates of split times between them considering a mutation rate of 4 × 10^−9^ mutations per base, per generation. The split times for BAL-NCE and BAL-IBE estimated with *fastsimcoal2* (S1I Fig) are indicated by red and blue triangles on the x-axes, respectively. The top panel shows ancestral changes in N_e_ within the groups plotted against time in years, when considering 1 generation per year. With *MSMC2* (**E**), 20 random sets of 4 strains were analyzed, which are all plotted, while with relate (**F**), all strains were analyzed jointly, hence a single line. The bottom panels show the RCCRs in BAL vs. NCE (solid lines) and IBE vs. BAL (dashed lines). Light blue shaded areas in the plots show ancient periods of glaciation according to MISs 2–4, 6, 8, 10, 12, 14, 16, and 18 [45], respectively, from left to right. The period of the LGM [46] is likewise indicated by the darker blue shade embedded in MIS2–4. The data underlying the graphs shown in this figure can be found at https://doi.org/10.5281/zenodo.7907435. BAL, Balkan; IBE, Iberian; LGM, last glacial maximum; MIS, marine isotope stage; NCE, Northern Central European; PC, principal coordinate; PGD, pairwise genetic distance; RCCR, relative cross coalescence rate.

As a first step in these comparisons, we resequenced a set of 488 *C*. *hirsuta* strains and investigated patterns of genetic structure based on 5,336,586 biallelic high-quality SNPs. The geographic distribution of our panel is centered on Western Europe but also includes a small number of strains from the United States of America, New Zealand, Australia, and Japan (S1 Table). We used the software *ADMIXTURE* [44] to estimate the most likely number of ancestry groups and for each strain the proportion of ancestry from each group (Figs 1A, 1B and S1A). The results of this analysis revealed the presence of 3 genetically differentiated groups: (1) the Iberian (IBE) ancestry group; 2) the Balkan (BAL) ancestry group; and (3) the Northern Central European (NCE) ancestry group. IBE included mainly strains that were collected from the Iberian Peninsula but also from France, Great Britain, the Netherlands, Ireland, USA, and New Zealand (Fig 1B). Strains with substantial ancestry from IBE were also found to be widespread in the Macaronesian archipelagos of Madeira and the Azores. Strains from BAL were predominantly sampled in Croatia and Austria, but admixed strains with substantial ancestry from BAL were widely distributed over the European continent, Madeira, Ethiopia, Japan, and the USA. NCE was mainly composed of strains sampled from Europe between latitudes 50° and 60° north. *ADMIXTURE* results also indicated a large number of strains with shared ancestry from BAL and NCE indicating gene flow, while IBE appeared more isolated in this respect. Those findings were corroborated by a principal component analysis (PCA) in which nonadmixed individuals from the 3 populations represented well-differentiated genetic groups, while admixed strains were mainly observed between BAL and NCE (S1B Fig).

In *A*. *thaliana*, a set of genetically differentiated strains (so-called relicts) have been identified based on the examination of distributions of pairwise genetic distances (PGDs) between individual strains [17]. We conducted a similar analysis to compare the genetic differentiation between populations in our dataset to the reported differentiation between relicts and nonrelicts in *A*. *thaliana*. Unlike *A*. *thaliana*, for which this distribution has a single major mode around 0.475% (Fig 3A in [17]), PGDs in *C*. *hirsuta* display 2 major modes around 0.4% and 0.8% (Fig 1C). The center of the second mode is above the highest values observed for *A*. *thaliana* [17], and it reflects pairwise comparisons between IBE and non-IBE strains (Figs 1D, S1C and S1D). Notably, the Iberian *C*. *hirsuta* relict-like group shows broader distribution outside the Iberian Peninsula than its *A*. *thaliana* counterpart (S1E Fig). This observation suggests that *C*. *hirsuta* IBE relicts may have had higher potential to colonize diverse European environments than their *A*. *thaliana* counterparts.

We further compared ancestry group–specific patterns of diversity between *C*. *hirsuta and A*. *thaliana* by calculating nucleotide diversity, Tajima’s D, and *F*_ST_ values in the genetic groups described for both species (S2 Table). We found that in *C*. *hirsuta*, IBE is the most diverse followed by BAL and NCE. The nucleotide diversity in IBE (0.14%) is similar to the one observed in *A*. *thaliana* strains belonging to the Iberian group (relicts and nonrelicts). The lower diversity in NCE is consistent with the effects of recent demographic processes accompanying the colonization of northern latitudes by Southern European lineages after the last glacial maximum [46] and is further confirmed by a slower linkage disequilibrium (LD) decay in NCE compared to other populations from *C*. *hirsuta* and *A*. *thaliana* (S1F Fig). Tajima’s D values in *C*. *hirsuta* are positive and more positive than in *A*. *thaliana* (with the exception of IBE) and potentially result from more pronounced population size bottlenecks (S2 Table), which may also be responsible for the high variance in Tajima’s D in NCE. *F*_ST_ values indicate that the genetic differentiation between IBE and the other groups in *C*. *hirsuta* (*F*_*ST*_ = 0.55) is 45% larger than the highest genetic differentiation observed between *A*. *thaliana* relict and nonrelict populations in the 1001 Genomes Project (*F*_*ST*_ = 0.38; S2 Table). Thus, the genetic differentiation of IBE versus non-IBE *C*. *hirsuta* is more substantial than relict versus nonrelict *A*. *thaliana*, while NCE presents a bottleneck signature not found in geographically equivalent *A*. *thaliana* populations.

To understand the nature of the evolutionary processes that shaped patterns of variation in *C*. *hirsuta*, we conducted a series of demographic inference analyses aiming at estimating changes in population sizes, the ages of population divergences, and the level of gene flow between populations. We estimated piece-wise constant distributions of population sizes and cross-coalescence rates through time for IBE, BAL, and NCE using the software *relate* [47] and *MSMC2* [48–50] (Figs 1E, 1F, S1G and S1H). In both cases, results indicated that the oldest event in the history of our sample is the divergence between the ancestral lineages of IBE and the one ancestral to both BAL and NCE. The divergence between BAL and NCE, on the other hand, occurred more recently and was followed by a pronounced reduction in effective population size of NCE, which might reflect the colonization of higher latitudes (Fig 1E and 1F). To obtain point estimates for those divergence times, we conducted model choice and parameter optimization for 4 alternative demographic scenarios using the maximum-likelihood optimization method implemented in *fastsimcoal2* [51,52]. We assumed the population tree suggested by the *relate* and *MSMC2* analyses to be correct and focused our model choice analysis only on the absence or presence of gene flow and population size bottlenecks. Our best model (S1I Fig and S3 Table) was characterized by gene flow between all populations and population size bottlenecks in IBE and NCE. Estimates for the 2 divergence times (indicated by triangles in Fig 1E and 1F) were in line with results obtained using *relate* and confirmed an ancient divergence of ancestral IBE lineages (approximately 534,000 years) and a more recent split between NCE and BAL (approximately 16,000 years). Also, ancestral and present population size estimates largely agreed with the estimates from *relate* and *MSMC2*, and all methods identified a reduction in size for NCE following its divergence. Overall, our observations indicate the presence of a relict group in Iberia, thus highlighting striking parallelism in spatial population structure with *A*. *thaliana* despite the estimated divergence time of the 2 species being over 25 million years ago [53]. Furthermore, the recent colonization of higher latitudes by NCE suggests that potential associated adaptive events, which underpinned associated shifts in photoperiod and seasonality, might be detectable at the genomic level in this population [54,55].

### GWAS reveals heterochrony as a major determinant of leaflet number in *C*. *hirsuta*

**Fig 2:**
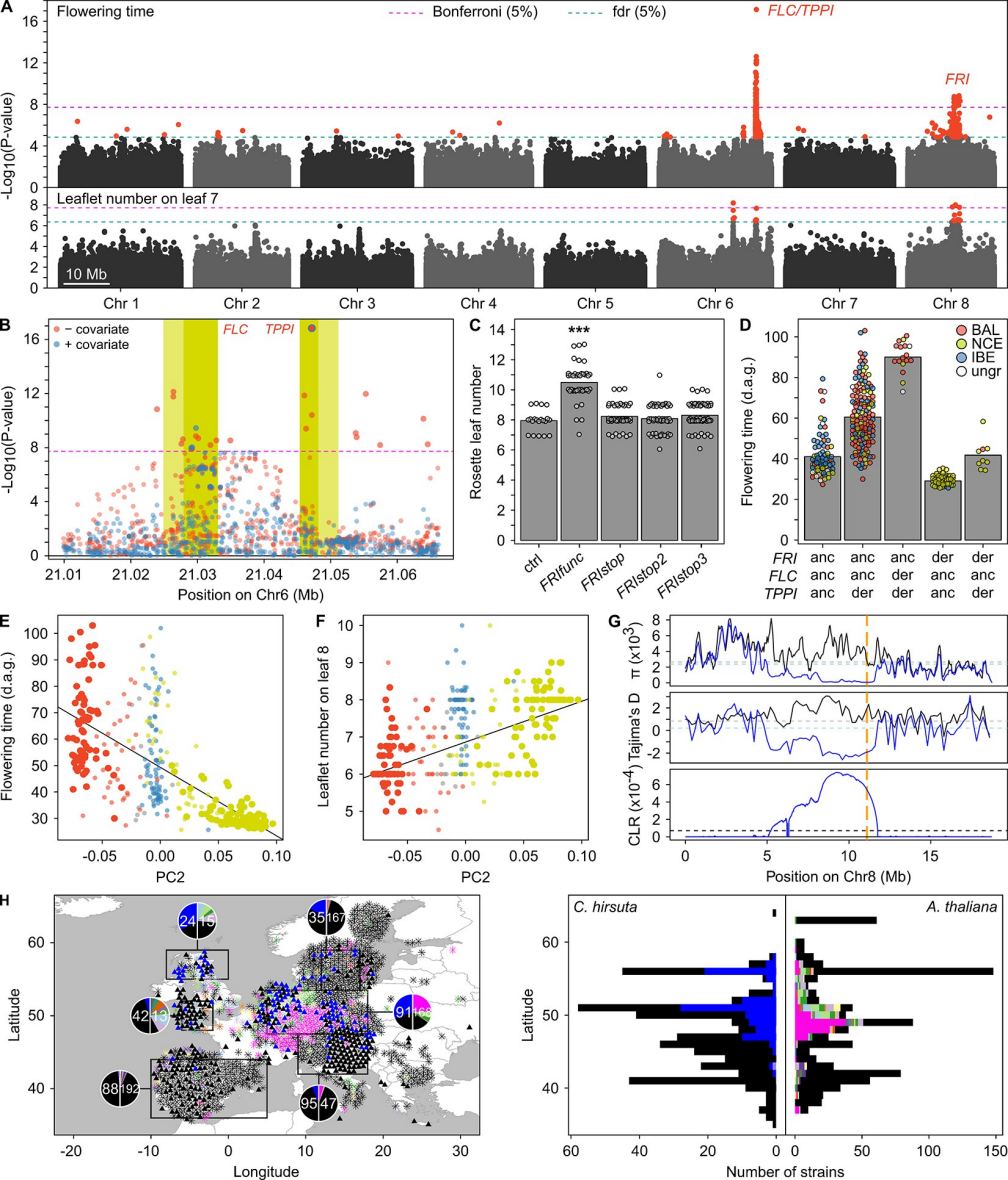
Selection for accelerated developmental progression in Northern and Central Europe. (**A**) GWAs for flowering time and leaflet number on leaf 7 using 352 *C*. *hirsuta* strains. The negative log base 10 transformed *P* values for association tests of individual SNPs are plotted against physical position on the 8 chromosomes. Horizontal dashed lines show thresholds of significance with correction for multiple testing according to Bonferroni (magenta) and fdr (cyan; for both α = 0.05). SNPs with transformed *P* values above the fdr threshold are shown in red, and others in gray. Two regions with strongly associated SNPs were detected on chromosomes 6 and 8 that contained the candidate genes *FLC/TPPI* and *FRI*. (**B**) Close-up view of the locus with the strongest associations showing GWAS for flowering time with the most significant SNP in *TPPI* used as covariate. Forward regression using a multilocus mixed model GWAS indicated that the highly significant association on chromosome 6 consisted of 2 independent associations. Yellow areas indicate the 2 candidate genes *FLC* (left) and *TPPI* (right) where the lighter shades indicate the promoter region (−3,000 bp) and the darker shades indicate the ORF. GWAS without covariates is shown in red, and with the SNP indicated by the blue encircled red point in *TPPI* as a covariate in blue. This result revealed significant associations for SNPs in the first intron of *FLC* that were independent of the associations for SNPs linked to *TPPI*. The associated SNPs in *FLC* shown in blue were the most significant genome-wide in this analysis. (**C**) Functional validation of 3 distinct truncated *FRI* alleles that exist within predominantly European samples of *C*. *hirsuta*. In contrast to a significant increase in rosette leaf number in plants transformed with a full-length *FRI* allele, the truncated *FRI* alleles showed no effect (Dunn test with Bonferroni adjusted *P* value, ***: *P* value < 0.001). One of the 3 alleles was found at high frequency (*FRIstop*) and exclusively in NCE strains (see also S2B Fig). (**D**) Flowering time in DAG until anthesis for all genotype combinations at the 3 candidate genes identified by GWA. The genotypes at the representative SNPs for each gene are shown as either anc or der. The bars indicate the mean flowering time, and the points show the individual observations for each strain. Points are colored according to the ancestry group of strains (Fig 1A). (**E**, **F**) Correlation between North–South genetic differentiation (PC2 in S1B Fig) and flowering time (**E**) as well as leaflet number on leaf 8 (**F**). The points are observations for individual strains colored according to their ancestry group (Fig 1A) such that strains with ancestry in only 1 group are shown in darker shades vs. lighter shades for admixed. The lines show linear models fitted to the data from the BAL and NCE populations (*P*<0.001, R2 = 0.54, r = -0.73, Fig 2E; *P*<0.001 R2 = 0.38, r = 0.62, Fig 2F). Large dots show non admixed samples in those two populations. (**G**) Evidence for a selective sweep at the *FRI* locus (see also S2 Fig). A sliding window analysis of nucleotide diversity (π, top), Tajima’s D (middle), and CLR calculated by *SweepFinder2* [59] (bottom) is shown for chromosome 8. The analyses were performed separately in strains with the *FRIstop* (blue) and the *FRIfunc* (black) alleles from the NCE group (Fig 1A). Note how the region, which includes the *FRI* locus (orange dashed line) displays reduced π, reduced Tajima’s D, and high CLR, consistent with a selective sweep, exclusively in strains with *FRIstop*. The horizontal dashed lines in the top and middle panels indicate the genome-wide averages for the respective groups in blue or gray, and in the lower panel the horizontal dashed line indicates the threshold (α = 0.05) derived from neutral simulations using our best demographic model. (**H**) The geographic distribution of full-length and truncated *FRI* alleles on the map and their projection on latitude in *A*. *thaliana* (*) and *C*. *hirsuta* (triangle) exhibited high similarity. The colors represent distinct truncated *FRI* alleles. The rectangles represent areas of high sampling density for both species. The pie charts show the proportion of functional and nonfunctional alleles in *C*. *hirsuta* (left) and full-length and truncated alleles in *A*. *thaliana* (right). The total number of strains inside the respective rectangles is shown inside the pie chart. The histograms on the right side show functional/full-length (black) and nonfunctional/truncated *FRI* alleles (different colors represent different truncated alleles) along the latitude. *FRIstop* is the major truncated *FRI* allele. Note that only one of all mainland European *C*. *hirsuta* strains harbors *FRIstop2* and none *FRIstop3*. Map layers were made with Natural Earth and [142]. The data underlying the graphs shown in the figure can be found at https://doi.org/10.5281/zenodo.7907435. anc, ancestral; BAL, Balkan; CLR, composite likelihood ratio; DAG, days after germination; der, derived; fdr, false discovery rate; *FLC*, *FLOWERING LOCUS C*; *FRI*, *FRIGIDA*; GWA, genome-wide association; IBE, Iberian; NCE, Northern Central European; ORF, open reading frame; SNP, single nucleotide polymorphism; *TPPI*, *TREHALOSE-6-PHOSPHATE-PHOSPHATASE I*.

Having established the above framework for understanding demographic events that shaped European *C*. *hirsuta* genetic diversity, we next sought to link phenotype with genotype in corresponding germplasm. To this end, we measured flowering time and leaflet number in 352 strains from our diversity panel. We observed considerable phenotypic variation after accounting for population structure (S2A Fig). We used genome-wide association (GWA) analysis to identify genetic variants associated with flowering time and leaflet number. Two highly significant associations were detected for flowering time on chromosomes 6 and 8, respectively (Fig 2A), and those same loci were also found to contain SNPs strongly associated with leaflet number on the later leaf nodes (Fig 2A). A closer inspection of the associations on chromosome 6 revealed 2 candidate genes within a 20-kb region: *FLC* and the *C*. *hirsuta* orthologue of *trehalose-6-phosphate phosphatase I* (*TPPI*). *FLC* influences flowering time and leaf development in both *A*. *thaliana* and *C*. *hirsuta* [25,56,57]. TPPI catalyzes the dephosphorylation of trehalose-6-phosphate, the concentration of which influences flowering time and heterochronic shoot development in *A*. *thaliana* [58]. The 15 most significantly associated SNPs on chromosome 6 were found in close proximity to *FLC* and *TPPI* and 6 of them inside their open reading frames or promoter regions. Multilocus GWAS showed that SNPs in both genes had independent effects, indicating that both contribute to phenotypic diversity in our strain panel (Fig 2B).

**Fig 3:**
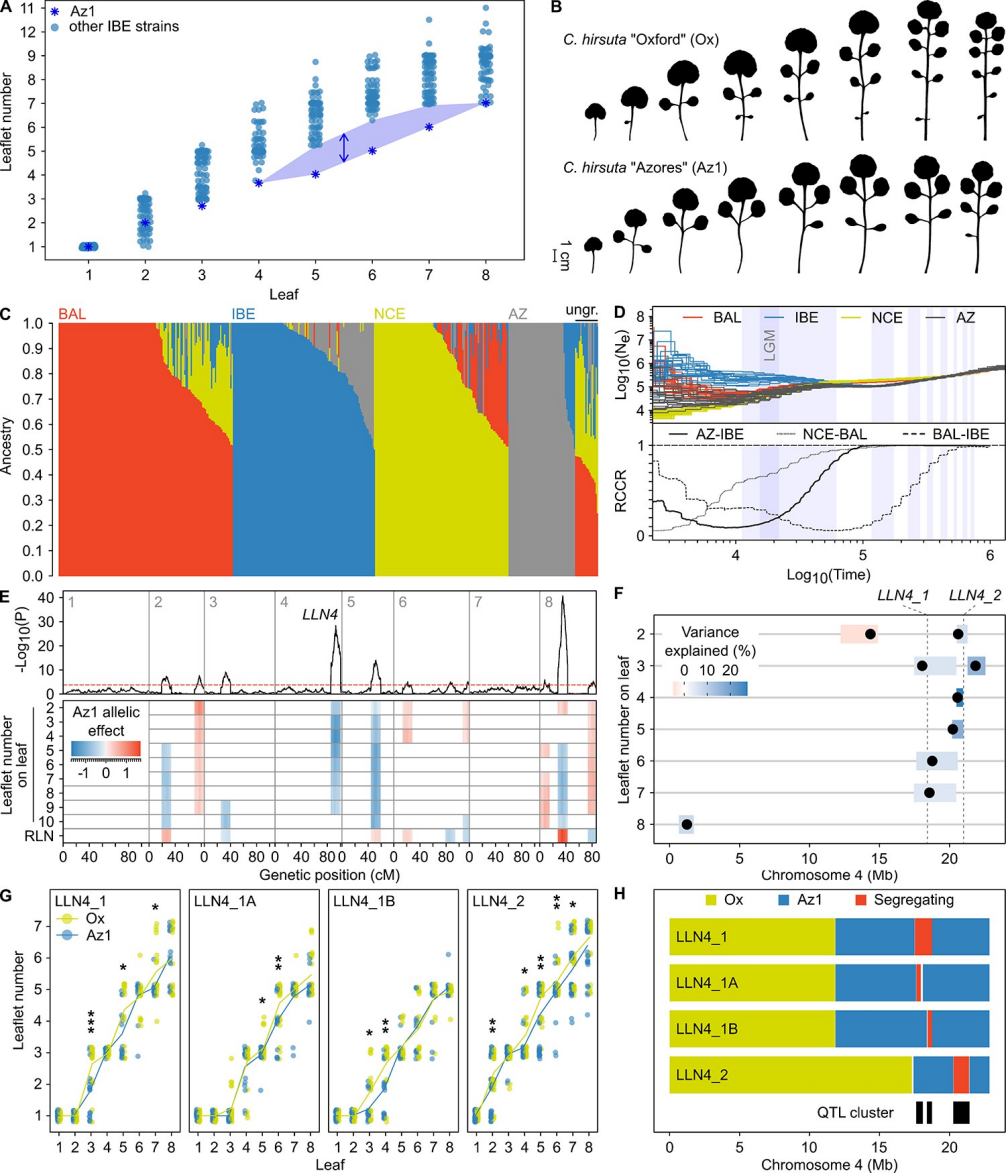
A QTL cluster on chromosome 4 contributes to low leaflet number in the Azorean *C*. *hirsuta* strain. (**A**) Leaflet number progression from the first to the eighth leaf indicates a strong deviation of the Az1 strain from other strains of the IBE group (see also S3A Fig). The leaflet number per leaf node of IBE strains is shown by blue points with that of Az1 shown by blue asterisks. The shaded area highlights the difference of Az1 compared to other IBE strains. (**B**) Representative silhouettes of the first 8 rosette leaves of 4 week-old *C*. *hirsuta* Ox and Az1 strains grown in long days, showing the lower leaflet number of the latter. (**C**) *ADMIXTURE* analysis with 421 *C*. *hirsuta* strains remaining out of 753 after filtering for close relatedness. The number of ancestry groups that best fit the data was found to be 4. Each vertical bar represents a strain where the colors indicate admixture proportions for the 4 ancestry groups. Strains were assigned to the ancestry group for which the proportion of ancestry was at least 0.5. The 3 clusters from Fig 1A were found again, and strains with maximum ancestry in the additional cluster were exclusively from the AZ. Strains with ancestry lower than 0.5 in all clusters are indicated as “ungrouped” on the right side of the figure. (**D**) Piecewise constant effective population sizes (N_e_) of the 4 ancestry groups from Fig 3C using *MSMC2*, and estimates of split times between them considering a mutation rate of 4 × 10^−9^ mutations per base, per generation. The top panel shows ancestral changes in N_e_ considering 1 generation per year. Colors indicate ancestry groups according to Fig 3C. Twenty random sets of 4 strains were analyzed, which are all plotted individually. The bottom panel shows the RCCRs in AZ vs. IBE (solid line), IBE vs. BAL (long dash line), and NCE vs. BAL (short dash line). Light blue shaded areas in the plots show ancient periods of glaciation according to MIS 2-4, 6, 8, 10, 12, 14, 16, and 18 [45], respectively, from left to right. The period of the LGM [46] is likewise indicated by the darker blue shade embedded in MIS 2–4. (**E**) Multiple trait QTL mapping of leaflet number from the first to the 10th rosette leaf and total RLN (a proxy for flowering time) in the Ox x Az1 RIL population. The negative log base 10 transformed *P* values of a composite interval mapping scan are plotted against position on the linkage groups of the chromosomes indicated in the top left corners of the upper panel. The horizontal dashed red line indicates the threshold of significance (α = 0.05). Significant allelic effects for each QTL on each trait are shown in the lower panel where red and blue colors indicate the direction, and the shade the magnitude of the effect according to the legend in the top left. (**F**) Multiple QTL models for leaflet number on different leaf nodes on chromosome 4. QTL detected using MQM mapping for the traits indicated on the y-axis are shown by black dots, and the 1.5 LOD intervals are indicated by shaded regions. The color of the 1.5 LOD intervals indicates the variance explained by the QTL according to the legend above the figure. Note that the direction of effect of both QTL agree with the parental differences in leaflet number (i.e., Az1 had lower leaflet number than Ox). (**G**) Leaflet number of HIFs segregating for different genomic regions of chromosome 4. Leaflet numbers of lines homozygous for Ox or Az1 alleles are shown in yellow and blue, respectively. Vertical bars indicate the standard errors of the means, and the points show the leaflet numbers of individual replicates. Significant differences in leaflet number for specific leaf nodes are shown as: *, *P* ≤ 0.05; **, *P* ≤ 0.01; ***, *P* < 0.001. Note that plants with Az1 alleles of HIFs LLN4_1A and LLN4_1B both show reduced leaflet number, but on earlier or later leaf nodes, respectively. By contrast, plants with Az1 alleles in HIF LLN4_1, which carries a larger introgression including *LLN4_1A* and *LLN4_1B*, show reduced leaflet number on earlier and later leaf nodes. (**H**) Graphical representation of the genotype of chromosome 4 in HIFs. Yellow and blue colors indicate homozygous Ox and Az1 alleles, respectively, while segregating regions are colored in red. Map positions of the 3 distinct QTL found in this region are depicted as black boxes. The data underlying the graphs shown in the figure can be found at https://doi.org/10.5281/zenodo.7907435. AZ, Azores; Az1, Azores1; BAL, Balkan; HIF, heterogeneous inbred family; IBE, Iberia; LGM, last glacial maximum; MIS, marine isotope stage; NCE, Northern Central Europe; Ox, Oxford; QTL, quantitative trait locus; RCCR, relative cross coalescence rate; RIL, recombinant inbreeding line; RLN, rosette leaf number.

The significantly associated region on chromosome 8 harbored a block of extended LD that contained *FRIGIDA* (*FRI*), a well-studied gene controlling flowering time through the activation of *FLC* expression [60,61]. *FRI* thus emerged as a candidate for determining natural variation in flowering time and leaflet number in *C*. *hirsuta*. We found 3 independent polymorphisms predicted to truncate the *FRI* protein. *C*. *hirsuta* Oxford (Ox) lines constitutively expressing the coding sequence (CDS) of any truncated *FRI* allele driven by the *Ubiquitin10*-promoter (*UBQ10*) did not show increased rosette leaf number (RLN), a proxy for flowering time, while lines expressing a nontruncated CDS (*FRIfunc*) increased RLN by 20%, confirming the functional significance of the truncations (Fig 2C). Of the 3 alleles, only one (*FRIstop*) occurred at high frequency and could therefore be responsible for the association on chromosome 8. This allele is specific to NCE only and was present in 45 out of 57 nonadmixed strains (S2B Fig). These observations coupled with QTL mapping in recombinant inbreeding line (RIL) populations derived from biparental crosses ([25] and Fig 3E) indicate that the *FRI/FLC* module is a major determinant of natural variation for flowering time and leaflet number progression in *C*. *hirsuta* (S2D Fig).

### Selection of a *FRIGIDA* loss-of-function mutation highlights similarities and differences in adaptation of *C*. *hirsuta* and *A*. *thaliana*

In the candidate genes *FLC* and *TPPI* from GWA analyses, the derived allele was associated with increased flowering time compared to the ancestral allele and vice versa for *FRI* (Fig 2D). Strains harboring all 3 alleles that reduced flowering time belonged to the NCE population and indeed displayed the lowest average and variance in flowering time (Fig 2D). Furthermore, the principal component (PC) accounting for the differentiation between BAL and NCE (S1B Fig) negatively correlated with flowering time and positively with leaflet number (Fig 2E and 2F). Considering that *FRI* alleles with reduced function have been previously identified as targets of natural selection in *A*. *thaliana* [29,43,62–66], we hypothesized that the functional variation we identified at *FRI* may have allowed *C*. *hirsuta* to recently adapt to northern European climatic conditions. To test this hypothesis, we conducted a genome-wide scan for signatures of positive selection using the program *SweepFinder2* [59], which uses a composite likelihood ratio statistic to identify areas whose polymorphism patterns deviate from neutral expectations. We found that the *FRI* locus contained the most significant composite likelihood ratio values after controlling for confounding demographic effects using our best demographic model for the NCE population (Figs 2G and S2E). This region also displayed low nucleotide diversity (π) and Tajima’s D compared to the genomic background indicating reduced genetic variation and an excess of low-frequency alleles, the hallmark of a recent selective sweep [67] (Figs 2G and S2E–S2G). Notably, the *FRI* locus is located on the distal extreme of the pericentromeric region of chromosome 8 [53] (S9 Table). Consequently, the selective sweep spans genomic regions with very different recombination rates per generation and nucleotide (*r*), ranging from 4.7 × 10^−10^ to 6.6 × 10^−9^ within and flanking the pericentromeric region. We hypothesized that the reduced *r* in the pericentromeric region could explain why the selective sweep extends further in the proximal pericentromeric area of the *FRI* locus, in strains with the *FRIstop* allele. We tested this hypothesis by simulations and found that the sweep profile is consistent with a hard sweep of a single strongly selected beneficial mutation that occurred after the divergence time between lineages of NCE and BAL (S2H Fig). We then investigated the geographic distribution of *FRI* loss-of-function alleles in *A*. *thaliana* and *C*. *hirsuta*. Given that both species have similar life cycles and face, at least in part, similar ecological challenges, we reasoned that if parallel evolution of *FRI* loss-of-function alleles contributed to local adaptation, this allele should exhibit similar geographic distributions in the 2 species. To test this idea, we exploited genome resequencing data from 1,115 *A*. *thaliana* and 426 *C*. *hirsuta* strains from Europe. We found that *FRI* loss-of-function alleles showed a similar geographical distribution in both species with high frequencies in Central Europe and Northern Britain, low frequency in Sweden, and even lower frequency in Southern Europe (Fig 2H). This observation, together with the evidence for a selective sweep, is consistent with *ChFRIstop* evolving adaptively in this range. Like in *A*. *thaliana*, environmental conditions such as sufficient precipitation and relatively short winters in these latitudes would allow for both summer annual and winter annual behavior [35,68]. Fast cycling lines harboring *FRI* loss-of-function alleles would be more likely to complete a full cycle before the onset of winter when germinating in late summer and, therefore, produce more offspring [69]. A striking difference between the 2 species was the number of distinct *FRI* loss-of-function alleles. We detected in total 3 distinct truncated *FRI* alleles in *C*. *hirsuta*, all of which showed a loss-of-function phenotype in transgenics experiments (Fig 2C). Contrastingly, we found 29 different truncated and likely loss-of-function *FRI* alleles in the entire set of *A*. *thaliana* strains (S2I Fig and S4 Table). The most abundant loss-of-function allele in *A*. *thaliana* [60] occurred in 40% of the European *A*. *thaliana* strains that harbored *FRI* loss-of-function alleles (112 out of 280 strains), while *FRIstop* was found in 98.2% of the European *C*. *hirsuta* strains with loss-of-function alleles (111 out of 113 strains).

In summary, our data indicate evolution of the same genetic mechanism of reduced *FRI* activity in both *C*. *hirsuta* and *A*. *thaliana* to confer fast cycling during colonization of Northern and Central Europe after the last glacial maximum. This conserved mechanism, even in a genetically complex trait like flowering time [70,71], highlights how evolution reuses specific genetic modules in a predictable way [5]. Within this framework of conservation, which indicates the necessity of fast cycling for adaptation after a glacial maximum, an important difference is that a different number of loss-of-function alleles appears to have supported evolution of fast cycling in the 2 species. This difference might be traced to differences in demography and genomic architectures, which led to a repeated selection of multiple alleles in *A*. *thaliana* versus what is essentially a single event in *C*. *hirsuta* (see Discussion).

### Evidence for local adaptation in the Azores archipelago mediated by the transcription factor SPL9

The *FRI/FLC* module studied above affects flowering time concomitantly with the rate of vegetative development including node-dependent changes in leaflet number. This finding highlights that age-dependent progression of leaflet number provides an attractive trait for monitoring the progress of developmental time. To gain further insight into natural variation for heterochronic mechanisms in *C*. *hirsuta* and their possible role in local adaptation, we evaluated variation in leaflet number relative to flowering time. In the GWA panel, cumulative leaflet number of the first 8 rosette leaves and flowering time correlated negatively (*P* < 0.001, cor = −0.34, Pearson’s correlation). We reasoned that if heterochronic processes have a central role in shoot morphological variation in this species, they might also underlie variation between strains that do not differ in flowering time. This would be similar to the situation in snapdragon, where genetic control of age-dependent leaf shape variation is independent of flowering time [72], and it would also be consistent with findings that vegetative phase change and reproductive competence are, to some degree, genetically separable in *A*. *thaliana* [73,74]. We also postulated that if such heterochronic variation independent of flowering time exists, then its biogeographical distribution might help us understand its adaptive relevance. We noted that among the IBE strains in our GWA panel, flowering time and leaflet number did not correlate (S3A Fig). On average, the IBE strains produced the highest leaflet number compared to NCE and BAL. Interestingly, one IBE strain, Azores1 (Az1), originating from the Azorean island Faial, produced a conspicuously low number of leaflets (Fig 3A and 3B) despite being early flowering and thus, in contrast to the accelerated heteroblastic progression, typically found in early flowering strains [25]. We hypothesized that this strong shift in leaflet number with respect to other IBE strains might be a consequence of local adaptation in the Azores.

**Fig 4:**
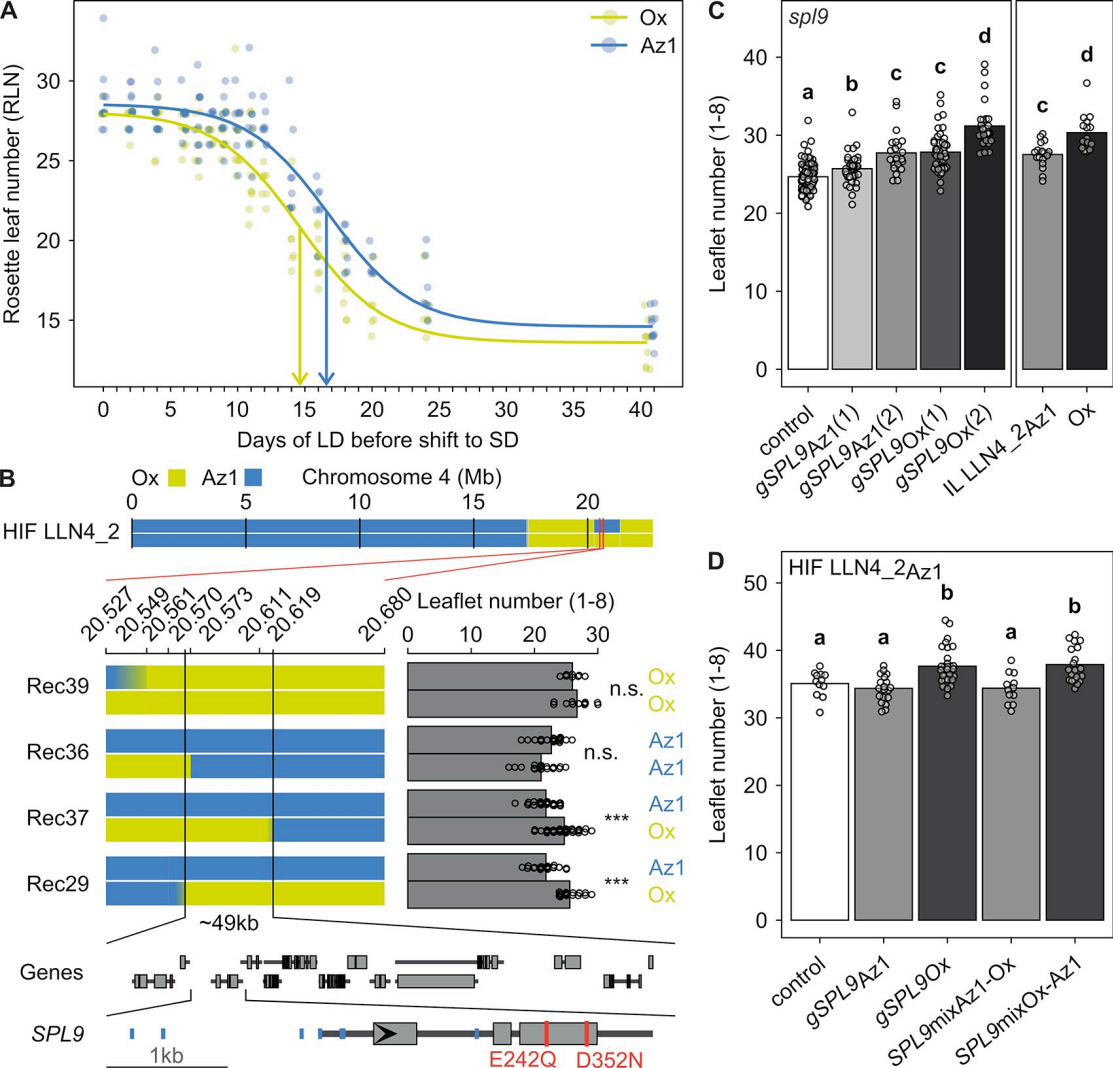
A missense polymorphism in *SPL9* underlies leaflet number QTL *LLN4_2*. (**A**) Photoperiod shift experiment showing that the Az1 alleles at the QTL *LLN4_2* delay the juvenile-to-adult phase transition. Plants of a HIF homozygous for Ox (yellow) or Az1 (blue) alleles at the *SPL9* locus were shifted from flowering inducing long photoperiod to a noninductive short photoperiod. The RLN of the plants is plotted against the time spent in long photoperiod. Points show the RLN of individual plants, while the lines show a logistic model fitted to the data. The inflection point of the model is indicated by vertical arrows on the x-axis. (**B**) Fine-mapping of the leaflet number QTL *LLN4_2*. The genotype information of the HIF LLN4_2 is shown in the top panel. The graphical genotypes of the homozygous progeny of 4 different recombinant lines segregating in the *LLN4_2* genomic region are shown below including the positions (Mb) of the genetic markers in the top axis. The bar chart on the right shows the number of leaflets produced on leaves 1 through 8 for the respective genotypes on the left. The bars show the mean leaflet numbers, and the points the leaflet numbers of the individual replicates. Kruskal–Wallis tests were performed to test for leaflet number differences between the 2 homozygous progenies of the same heterozygous recombinant: *** *P* < 0.001, n.s. nonsignificant. On the right side, the genotype at the *LLN4_2* locus (Ox or Az1) inferred from the phenotype of each line is depicted. The *LLN4_2* fine-mapped region of 49 kb contains 14 genes shown in the lower part of the panel with wider rectangles indicating exons and narrow rectangles introns and UTRs. The region containing *SPL9* is expanded at the bottom with the 2 missense SNPs differing between Ox and Az1 colored in red and other SNPs in blue (see also S4A Fig). (**C**) Transgenic complementation of the *Chspl9* mutant with the genomic constructs of *SPL9Ox* (*gSPL9Ox*) and *SPL9Az1* (*gSPL9Az1*). The estimated copy number of the transgene is indicated in parentheses. As a control, the *Chspl9* mutant was transformed with an empty vector. Two copies of *gSPL9Ox* and *gSPL9Az1* could complement the phenotype to the level of Ox wt and IL LLN4_2Az1, respectively. Dots correspond to individual T2 transgenic plants derived from 27 independent T1 plants, and their mean and standard error for cumulative leaflet number on the first 8 leaves is shown by the bars. The compact letter display shows significant differences between genotypes according to a Dunn test with a Benjamin–Holm post hoc correction of the *P* values for multiple pairwise comparisons. (**D**) Allele swaps for the 2 *SPL9* missense SNPs differing between Ox and Az1. The line HIF_LLN4_2 homozygous for the Az1 allele at the *SPL9* locus (Fig 4A and 4B) was transformed with the genomic constructs shown in Fig 4C, and with 2 additional chimeric genomic constructs carrying the Ox and Az1 alleles, or the Az1 and Ox alleles for the SNPs (*SPL9*mixAz1_Ox and *SPL9*mixOx_Az1). The HIF was transformed with an empty vector as a control. Dots correspond to individual independent T1 transgenic plants, and their mean cumulative leaflet number on the first 8 leaves is shown by the bars. The compact letter display shows significant differences between genotypes according to a Dunn test with a Benjamin–Holm post hoc correction of the *P* values for multiple pairwise comparisons. The data underlying the graphs shown in the figure can be found at https://doi.org/10.5281/zenodo.7907435. Az1, Azores1; HIF, heterogeneous inbred family; Ox, Oxford; RLN, rosette leaf number; wt, wild type.

To test this hypothesis, we first explored the prevalence of the low-leaflet phenotype. We carried out denser sampling and sequenced 267 *C*. *hirsuta* strains from across the Azores archipelago and found that the low leaflet number occurred in approximately 24% of them (S3B Fig). *ADMIXTURE* analysis revealed a well-differentiated ancestry group specific to the Azores (AZ; Figs 3C and S3C) that included the Az1 strain. Demographic analyses using *MSMC2* and *relate* estimated that the AZ and IBE ancestry groups diverged at least 30,000 years ago, thus indicating that *C*. *hirsuta* populations on the Azores might have been locally adapting for a long period of time (Figs 3D and S3D). To determine the genetic basis of this low-leaflet phenotype, we performed QTL mapping of leaflet number and RLN in an RIL population derived from a cross between Az1 and the NCE strain Ox (Fig 3E and S6 Table). We detected 7 QTL affecting both RLN and leaflet number (Fig 3E). Five out of the 7 loci had opposite effects on leaflet number and RLN similar to the heterochronic effect described for *TPPI/FLC* and *FRI* (Fig 2A; [25]). The remaining loci included a highly significant Leaflet number QTL on chromosome 4 (*LLN4*) that strongly affected leaflet number but not flowering time (Fig 3E). Genetic dissection of *LLN4* with 4 heterogeneous inbred families (HIFs) identified a cluster of 3 closely linked QTL for leaflet number located between positions 17.39 Mb and 22.83 Mb, which are referred to as *LLN4_1A*, *LLN4_1B*, and *LLN4_2* (Figs 3F–3H and S3E and S6 Table). In agreement with the parental behavior and the detected effects at *LLN4*, Az1 alleles at each of these loci reduced leaflet number. The effects of these 3 QTL were also validated in introgression lines (ILs; S3F Fig), showing that the most distal locus, *LLN4_2*, had the strongest effect on leaflet number. To further characterize *LLN4_2*, we first tested whether its effect on leaflet number was heterochronic and independent of flowering time. To this end, we carried out a photoperiod shift experiment using near-isogenic lines (NILs), which is based on the attribute of juvenile plants to not respond to the flowering-inducing stimulus of long photoperiod [75]. We found that a line homozygous for the Az1 allele delayed the juvenile-to-adult phase transition by 1.97 days compared to a line homozygous for the Ox allele (Fig 4A). However, both lines only showed a small but statistically insignificant difference in RLN in both short and long photoperiods. We concluded that the effect of *LLN4_2* is heterochronic, affecting the timing of the juvenile-to-adult transition and, consequently, leaflet number, largely independent of the flowering transition.

To identify the gene underlying *LLN4_2*, we first fine-mapped it to a 49-kb region containing 14 predicted genes. Among these, the *C*. *hirsuta* orthologue of the transcription factor encoding gene *SQUAMOSA PROMOTER BINDING-LIKE 9* (*SPL9*; Fig 4B) emerged as the best candidate for underlying this QTL because of its role in the regulation of developmental timing in *A*. *thaliana* and potentially leaflet number in *C. hirsuta* [75–77]. Consistent with this idea, analysis of a *C*. *hirsuta* loss-of-function allele of *SPL9* (*Chspl9)* generated by CRISPR-Cas9 genome editing showed reduced leaflet number, similar to the phenotype of the Az1 strain (S4A Fig). To directly compare the effects of both *SPL9* alleles, we analyzed transgenic *Chspl9* lines carrying genomic constructs of *SPL9* from either Ox or Az1. The *SPL9Az1* was less able than the *SPL9Ox* allele to increase leaflet number compared to the *Chspl9* mutant background (Fig 4C), thus indicating that it is a weaker allele. To test if these differential effects might be caused by altered *SPL9* expression of the 2 alleles, we conducted RNA-seq analyses in 2 pairs of genotypes and did not find *SPL9* among the list of differentially expressed genes (S4B and S4C Fig). However, analysis of the predicted coding sequence revealed 2 missense SNPs (E242Q and D352N) that could potentially affect the function of SPL9 (Figs 4B and S4D). We evaluated the effect of each of the 2 SNPs in HIFs transformed with the parental genes as well as with 2 chimeric constructs differing in only one of the 2 missense SNPs, respectively. Phenotypic analyses of leaflet number showed that transgenic lines with the Az1 or Ox alleles for SNP E242Q behaved similar to those transformed with Az1 or Ox genomic constructs, respectively (Fig 4D). Therefore, we concluded that allelic variation at *SPL9* underlies *LLN4_2* and that the missense polymorphism E242Q contributes to the low leaflet number of the Az1 strain.

**Fig 5:**
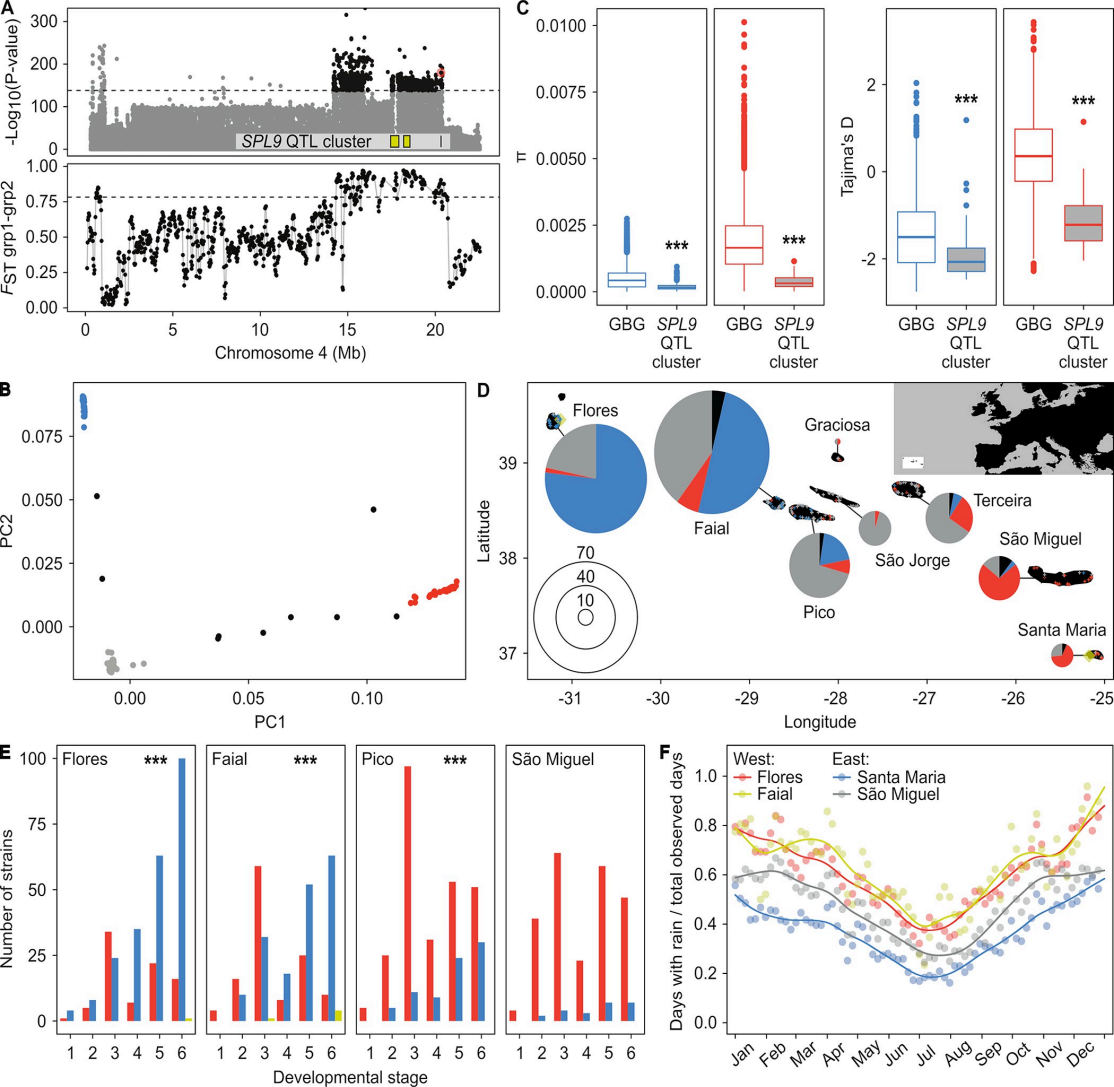
The *SPL9* QTL cluster as a driver of local adaptation in the Azores. (**A**) Signatures of selection at the *SPL9* QTL cluster. The upper panel shows a Manhattan plot with the *pcadapt* results for 753 *C*. *hirsuta* strains (see also S5B Fig). The negative log base 10 transformed *P* values for SNPs on chromosome 4 are plotted against their physical positions. The dashed horizontal line indicates the *P* value separating the 1,000 genome-wide most significant SNPs, among which 83.8% are located in the vicinity of the *SPL9* QTL cluster. The *SPL9* missense SNP E242Q that was found to be responsible for QTL *LLN4_2* is highlighted by a red circle. Yellow boxes in the lower part of the panel depict the location of QTL *LLN4_1A*, *LLN4_1B* and *LLLN4_2* (*SPL9*). The bottom panel shows a sliding window analysis along chromosome 4 of weighted *F*_ST_ between the 2 groups of strains from the Azores, grp1 and grp2, which were found as highly differentiated in the *SPL9* QTL cluster region (see also Fig 5B). The horizontal dashed line shows the genome-wide 95th percentile of weighted *F*_ST_. (**B**) PCA of the 838 outlier SNPs detected by *pcadapt* analysis that were located within the *SPL9* QTL cluster region, in 753 worldwide strains. Two groups of strains were highly differentiated from each other (grp1 –blue, grp2 –red) and from the other strains (gray and black). Strains with recombinations in the *SPL9* QTL cluster are shown in black. (**C**) Nucleotide diversity (π) and Tajima’s D in the region of the *pcadapt* peak surrounding the *SPL9* QTL cluster and the GBG outside of the peak for grp1 (blue) and grp 2 (red) strains from the Azores. (**D**) Geographic distribution of *C*. *hirsuta* strains within the Azores archipelago. The pie charts show the proportions of strains from the different groups in our sample colored according to Fig 5B (blue—grp1; red—grp2; black—recombinant in the *SPL9* QTL cluster; gray—others). Strains from grp1 and grp2 were exclusively sampled on the Azores and show a nonuniform distribution with highest frequencies in the east and the west, respectively. Map layers were made with Natural Earth and [142]. (**E**) Phenological field data of *C*. *hirsuta* plants from 4 islands along the west–east transect colored according to alleles for the functional missense SNP E242Q of *SPL9* (blue—Az1; red—not Az1; yellow—heterozygous;). Plants were classified for developmental stage from 1 (very young seedling) to 6 (advanced seed shedding). On Flores, Faial, and Pico, *SPL9Az1*-harboring strains were found in more advanced stages of development than strains carrying an alternative allele (*** *P* < 0.001, Kruskal–Wallis). (**F**) Weather station data from the Azores indicates reduced precipitation in the east when compared to the west. Data from 1970–2020 were analyzed in sliding windows of 11 days with 5-day stride and in each window the fraction of days with rain (> 0 mm) out of the total number of observed days was calculated. The fraction of days with rain in each window is shown by the points, and the lines show smoothing splines fitted to the data to reveal the trends. Note how on Santa Maria, in the east, from April until October, the fraction of days with rain is as low or lower than the lowest annual value for Flores and Faial in the west. The data underlying the graphs shown in the figure can be found at https://doi.org/10.5281/zenodo.7907435. GBG, genomic background; PCA, principal component analysis; QTL, quantitative trait locus; *SPL9*, *SQUAMOSA PROMOTER BINDING PROTEN-LIKE 9*.

To test whether genetic diversity in *SPL9* and its linked QTL which we collectively refer to as *SPL9* QTL cluster might be maintained by adaptive processes, we first confirmed the phenotypic effect of the *SPL9Az1* allele in field grown plants on the Azores (S5A Fig) and then searched for signatures of natural selection using *pcadapt* on the 753 Azorean and Eurasian strains. Similar to *F*_ST_-based methods, *pcadapt* identifies genomic regions with extreme genetic differentiation [78]. However, unlike *F*_ST_-based methods, it requires no prior population assignment. This feature can be advantageous for identifying regions that evolve nonneutrally in the presence of complex population structure and admixture, as is the case in *C*. *hirsuta* populations [78] (Figs 1 and S1). These analyses showed that the *SPL9* QTL cluster overlapped with the most significant peak detected by *pcadapt* (Figs 5A and S5B) and that 83.8% of the 1,000 genome-wide most significant SNPs fell within a 6.4-Mb genomic interval including the *SPL9* QTL cluster. Therefore, SNPs in this genomic region displayed a stronger pattern of genetic differentiation than the genomic background, consistent with the action of positive selection [78]. We then explored the genetic structure responsible for the *pcadapt* peak in the *SPL9* QTL cluster by performing PCA using only the SNPs within the interval that were above the threshold (Fig 5B). Two groups of 103 and 50 strains from the Azores showed stronger genetic differentiation at the *pcadapt* peak compared to the genomic background (S5C Fig). Accordingly, these 2 groups showed *F*_ST_ values above the 95th percentile of the genome-wide background, at the *pcadapt* peak (Fig 5A). Additionally, both groups displayed significantly lower nucleotide diversity and Tajima’s D in the *SPL9* QTL cluster region than in the remaining genomic background (Fig 5C), providing additional evidence that the *SPL9* QTL cluster genomic region has evolved under positive selection.

To investigate possible drivers of selection at the *SPL9* QTL cluster, we explored geographic and climatic associations of the 2 Azorean haplogroups in this genomic location (Fig 5B). Both groups showed a strong geographic structure along the West–East longitudinal axis of the Azorean islands (Fig 5D), which was stable across sampling years (S5D Fig). Haplotypes carrying the low-leaflet *SPL9* allele dominated in the West (grp1), while the alternative allele of grp2 was prevalent in the East (Fig 5E). Notably, this west–east clinal distribution mirrors that of other lineages in the endemic Azorean flora (e.g., *Leontodon*, *Ranunculus*, and *Tolpis*), which is thought to reflect a climatic gradient between warm summers with punctuated precipitations in the West, and extended dry summers in the East, in a context of reduced annual variation in temperature and daylength in the entire archipelago compared to mainland Europe [78–82] (Figs 5F and S5E–S5G). Thus, population genomics and QTL analyses in *C*. *hirsuta* revealed potentially adaptive allelic variation in the key heterochronic transcription factor *SPL9*, which appears as a major driver of morphological differentiation across heterogeneous climatic conditions.

## Discussion

*C*. *hirsuta* and *A*. *thaliana* are both predominantly self-pollinating plants that belong to lineage I of the Brassicaceae [83] and share similar annual life cycles, geographic distributions, and general habitats. Thus, comparative demographic analyses provide excellent opportunities to evaluate the genomic consequences of macroclimatic cycles such as glaciations on European populations of these 2 related species. Our results show that European populations of *C*. *hirsuta* comprise 3 ancestry groups among which IBE and BAL/NCE likely diverged in different glacial refugia and are now part of a secondary contact zone in Western Europe similar to *A*. *thaliana* [84]. Like in *A*. *thaliana*, one of those clusters is mainly found in the Iberian Peninsula and displays strong differentiation from the 2 other ancestry groups found in the Balkan region and in Northern Central Europe. The split time between Iberian and non-Iberian populations obtained with *fastsimcoal2* was 319 kya (S3 Table) when using the same mutation rate as in [17] (see Materials and methods). This split time is comparable to that estimated for divergence of relict and nonrelict European lineages in *A*. *thaliana* [17], as well as to the most ancient divergence times reported for any lineages in *A*. *thaliana* [19]. This finding indicates that *C*. *hirsuta* lineages have been isolated from each other over several glaciation periods. The higher prevalence of *C*. *hirsuta* relicts in Iberia (68 out of 86 strains versus 22 out of 190 in *A*. *thaliana*) and their broader geographic distribution into Western Europe (Fig 1B) suggests a potential for adaptation to more diverse environments than their *A*. *thaliana* Iberian counterparts.

We used the genome-wide catalog of SNP variation in a Eurasian strain panel to link genotype to phenotype for flowering time and leaflet number. These analyses showed that both traits are pleiotropically controlled and that *FRI/FLC* and/or *TPPI* contribute to synchronize vegetative and reproductive development. *TPPI* encodes an enzyme predicted to dephosphorylate trehalose-6-phosphate yielding trehalose. This metabolite is believed to function as a carbon availability sensor that also influences flowering time in age-dependent pathways [58,85,86]. Thus, natural variation in *TPPI* might also affect carbon sensing, potentially linking metabolism and development in the context of natural phenotypic diversity.

The study of natural variation in *FRIGIDA* illustrates how the balance of demography and adaptation may have shaped the evolutionary history and polymorphism patterns in the 2 species. In terms of adaptation, truncated *FRI* alleles occur mainly in strains that colonized northern Europe after the last glacial maximum, a pattern consistent with local adaptation. The split time between the Balkan and the Northern Central European cluster (16 kya; Fig 1E and 1F), the bottleneck in the demographic model (S1I Fig), and the continuously diminishing effective population size detected with *MSMC2*/*relate* (Fig 1E and 1F) largely agree with a scenario in which lineages arising from a southern refugium colonized Northern Europe following the end of the last period of glaciation. This pattern parallels findings in *A*. *thaliana* and is in line with the view that both summer and winter annual life cycles are maintained in these latitudes [35,69]. However, the differences in the number and frequencies of loss-of-function alleles segregating in *A*. *thaliana* and *C*. *hirsuta* indicate that the evolution of fast cycling was probably also shaped by additional factors modulating the adaptive response at the genomic level. One such factor might be the distinctive explosive seed dispersal strategy of *C*. *hirsuta*, which could have facilitated the rapid spread of a few beneficial mutations and ancient haplotypes [23]. Such a process might involve increasing connectedness between isolated subpopulations, which can reduce the time it takes for an adaptive allele to spread [23,87]. Alternatively, the difference in recombination rates around *FRI* in both species (caused by a larger pericentromeric region in *C*. *hirsuta*) also influences the pattern of rapid cycling caused by *FRI* loss-of-function mutations in Central Europe. While in *A*. *thaliana FRI* is located near the distal end of the short arm of chromosome 4, in *C*. *hirsuta*, it is located in the pericentromeric region of chromosome 8. The extent of genetic hitchhiking in selective sweeps depends on the local recombination rate [88,89] and, consequently, low-recombining regions tend to accumulate more deleterious mutations [90]. Fixation of multiple independent beneficial alleles of *FRI* might therefore have been restricted in *C*. *hirsuta* due to a higher potential genetic load compared to *A*. *thaliana*, which limits the capacity to spread through the population. Thus, this case of parallel evolution of *FRI* based on extensive comparative evaluation of diversity patterns of 2 species along a similar geographic cline illustrates how the complex interplay of demography, natural selection, and genome structure shapes adaptive processes. Natural allelic variation in *FRI* leading to flowering time variation has also been uncovered in other Brassicaceae such as *Brassica napus* [91] and *Arabidopsis lyrata* [92], so once larger data sets for more species exist, it will be interesting to evaluate spatial patterns of allelic variation as we have done here for *C*. *hirsuta*.

Our extensive analysis of Azores *C*. *hirsuta* germplasm indicates that this island complex harbors considerable *C*. *hirsuta* diversity even though previous work had suggested that overall biodiversity in the Azores might be low and very few lineages showed patterns of adaptive radiations there [93]. One possibility to explain these findings is that this island complex represents an old refugium where *C*. *hirsuta* established from seeds introduced perhaps by vagrant birds [94] over long periods of time and well before large-scale human colonization of the Azores in 1449 CE and the first evidence for human settlers in 700 CE [95, 96]. Within this context, the geographically localized distribution of the *SPL9* QTL cluster coupled with evidence for positive selection indicates that the phenotypic differentiation we report here may be adaptive in the Western Azores Islands. This observation raises the question of what might be the environmental drivers of this distribution. Examination of climate variables shows that a major difference of the Western Azores relative to the East are the more frequent summer rainfalls in the West, punctuated by dry spells [97] (Figs 5F, S5D and S5E). Under these conditions, seeds that germinate after rainfalls in summer months would likely face transient drought periods that may challenge seedling establishment and survival. Previous work has linked *SPL* function and the juvenile state to drought tolerance [39], suggesting that the prolonged juvenility conferred by the *SPL9* QTL might help such summer seedling establishment at the face of transient drought. In line with this view, strains harboring the *SPL9* QTL cluster were abundant throughout the year and already developmentally advanced in November in the Western Azores (Fig 5E), which is consistent with their successful establishment during the summer. It is also possible that the reduced function of *SPL9Az1* and a slower juvenile-to-adult transition is part of a life history strategy that evolved in the more broadly permissive Western Azores environment relative to the more seasonal mainland Europe, where a more stringently controlled transition to the reproductively competent adult stage might be beneficial. Isolation of additional QTL underpinned by Az1 alleles coupled with field studies and physiological assays as well as development of statistical frameworks for jointly estimating demography and selection [98] will shed more light on these issues. As a starting point, we confirmed that the reduction in leaflet number by *SPL9Az1* that we characterized in this study is also present in field grown plants (S5A Fig), supporting the hypothesis that it is relevant in natural settings. Overall, our repeated recovery of heterochronically acting QTL that also show evidence for selection highlights the high significance of age-dependent developmental pathways for evolution of plant form [99]. One reason for this prevalence might be that by regulating multiple aspects of development and response to the environment concurrently, these pathways provide evolution with the opportunity to rapidly tinker with integrated traits and modify life history strategies, through changing frequencies of allelic variants of heterochronic regulators such as *SPL*, *FRI*, and *FLC*. The *SPL9* and *FRI* variants we find here are in coding rather than regulatory regions. Regulatory variation frequently contributes to morphological diversity owing to relatively low pleiotropy preventing detrimental effects on development and fitness [100]. In the case of *FRI*, this might reflect its key role in the flowering time genetic network upstream of *FLC*, which has been proposed to make it a potential hotspot for phenotypic evolution [101] and demographic factors, as discussed above. *SPL9Az1*, on the other hand, is a relatively weak allele whose effects could also be partially buffered by the potentially redundant paralogue *SPL15* [75]. These facts may contribute to its prevalence by limiting potential detrimental effects.

The *C*. *hirsuta* genetic and phenotypic variation as well as demographic history we report here, together with prior work on *A*. *thaliana*, illustrate how integration of population-level analyses with interspecific comparative studies can advance our understanding on how the interplay of adaptive mechanisms, historically contingent processes, and developmental constraints shape evolution. The finding that *C*. *hirsuta* shows endemic features in the Azores, despite being a cosmopolitan weed, indicates the importance of in-depth diversity studies broadly across species distribution ranges, including distribution edges, for conservation efforts and for understanding long-term evolutionary trends in different ecosystems. Interestingly, *A*. *thaliana* occurs only sporadically in the Azores where it was first found in 1974 [80,102] (S7 Table), while it is abundant in other Macaronesian islands [19]. Future comparative studies using the resources we report here coupled with field work will help understand the basis for these differences. Such studies will also deepen our understanding on how the interplay between stochastic and deterministic factors shape similarities and differences in the ecogeographic distribution and phenotypic diversity of related species. They should also contribute to understanding the effects of current climate change on biodiversity.

## Materials and methods

### Resource availability

#### Lead contact

Further information and requests for resources and reagents should be directed to and will be fulfilled by the corresponding author Miltos Tsiantis (tsiantis@mpipz.mpg.de).

### Materials availability

Plasmids and seed generated in this study have been deposited in relevant collections of the Tsiantis lab at the Max Planck Institute for Plant Breeding Research and will be distributed upon request. For natural strains, only seeds of the GWAS panel will be curated in the Tsiantis lab at the Max Planck Institute for Plant Breeding Research.

### Ethics statement

We have done due diligence to ensure that seed collections are compliant with the Nagoya Convention on Biodiversity. The relevant field permit numbers for collection of plant material in the Azores, Portugal are as follows: SAI-DRA-2010-550, SAI/DRA/2011/1426, SAI-DRA/2014/1193, and 17/2017/DRA, provided by the local authorities in the Azores, Secretaria Regional da Energia, Ambiente e Turismo, Região Autónoma dos Açores.

### Experimental model and subject details

S1 Table lists the origin of *C. hirsuta* strains used here. 

### Plant cultivation

Plants were cultivated at the Max Planck Institute for Plant Breeding Research in Cologne, Germany, or at the Department of Plant Sciences, Oxford University, Oxford United Kingdom. *C*. *hirsuta* seeds were stratified on wet soil or filter paper in the dark for 10 days at 4°C. Plants were grown either in climate-controlled chambers/cabinets or in the greenhouse. In the former, long-day conditions consisted of a cycle of 16 hours day and 8 hours night with temperatures of 20°C/18°C day/night, a light intensity of 200 μmol·m^−2^·s^−1^, and relative humidity of 70%; and short-day conditions with an 8-hour day/16-hour night cycle and a light intensity of 175 μmol·m^−2^·s^−1^ that were otherwise identical to long-day. In the greenhouse, temperatures were set to 20°C and supplemental lights were switched on during the day when the light intensity fell below 75 μmol·m^−2^·s^−1^. The approximate relative humidity in the greenhouse was 60%.

## Method details

### A strain panel for investigating *C*. *hirsuta* genetic diversity and demography

#### Whole genome sequencing

DNA was extracted from fresh leaves of 752 strains other than the reference strain from Oxford and 1 *Cardamine oligosperma* strain using a CTAB DNA extraction protocol or an automated KingFisher Flex system with associated chemistry (Thermo Fisher Scientific). Paired-end library preparation and short read sequencing was performed at the Wellcome Trust Centre for Human Genetics (WTCHG, Oxford, UK) or at the Max-Planck Genome Center (MPGC, Cologne, Germany) using Illumina HiSeq2000 or HiSeq3000 instruments (Illumina). Read-lengths varied and included 49, 100, 150, and 151 nucleotides across projects (see S1 Table for details). We aimed for a raw read depth of 20× and achieved a median of 20.28× across all samples (see S8 Table).

Long reads of the reference strain Ox [53] were generated using the Oxford Nanopore GridION X5 at a coverage of 80× (MPGC, Cologne).

#### Variant calling and QC

The reference sequence of *C*. *hirsuta* used in this study is provided in [53], at https://doi.org/10.5281/zenodo.7907435, and at http://chi.mpipz.mpg.de/assembly.html, either directly or through a link from GenBank (Biosample: SAMN02183597; Bioproject: PRJNA293154). Short read alignment and variant calling was performed using the *IMR-DENOM-IRISAS* pipeline [103]. Quality control of the called SNPs was performed by means of dideoxy sequencing. A total of 32,333 bp of double stranded sequence containing 333 called SNPs was produced for 84 loci in 8 resequenced strains and the Ox reference. The sequence of the latter was found to fully agree with the reference genome. In the 8 strains, 2,640 SNP alleles were validated according to SNP calling, and 6 were found to be different, rendering an error rate of 0.23%. The 6 erroneously called SNPs were located in 2 loci. For 1 SNP, the alternative allele was not found in any of the 4 strains that should have it. For the second SNP, 2 singletons in a single sequenced fragment were not found in 1 strain.

Various analyses depended on accurate accounts of polymorphic and nonpolymorphic sites in the resequenced genomes. Several common and strain-specific masks were applied to control for different sources of uncertainty in the genomic data. We masked the resequenced genomes where the read depth was less than 4 reads. Read-depths were extracted for the 8 chromosomes from the bam files containing the reads of each resequenced strain aligned to the reference genome, respectively, using *samtools mpileup* version 1.9. *IMR-DENOM* identified sequence motifs that were particularly diverged and called them as single variants with alleles longer than 1 but not necessarily of equal length. A consequence of recalling the variants by *IRISAS* was that all differences between both alleles for these motifs were called as SNPs. We therefore masked the regions spanning the variants in question in each strain at the position at which it was called plus the length of the longest allele. To control for uncertainty in called SNPs conferred by sequence ambiguity in the reference genome, we applied Heng Li’s SNPable regions method for all 100-mer subsequences of the reference genome as described here: http://lh3lh3.users.sourceforge.net/snpable.shtml. Any position where the majority of overlapping 100-mers did not map uniquely and without 1 difference was masked in all strains. The masks described above were applied to the variants recalled by *IRISAS* for all subsequent analyses such that the allele at a SNP was changed to unknown if that position was masked for the strain in question. The number of unmasked sites was considered to be the total number of nucleotides observed for each strain. The SDI files produced by *IRISAS* were transformed to plink files using *IRISAS*’ SdiToSnpPlink command. The total number of SNPs was 11,576,732 with a 1.461%, 4.382%, 15.14%, and 12.49% first quantile, median, third quantile, and mean number of missing calls per SNP.

### Population structure and demographic analyses

#### Pairwise genetic distances and hierarchical clustering

PGDs were computed as in [17] from the number of detected SNPs between each pair of 488 strains and their genomic masks. The former was considered the number of differences, and this was divided by the total number of positions in the genome where neither strain was masked as the total number of observed sites. Principal coordinates analysis and hierarchical clustering of the resulting PGD matrix were performed in R using the functions *cmdscale* and *hclust*, respectively, to identify and visualize the major groups of distantly related strains. The results of hierarchical clustering were plotted as dendrogram using iTOL [104] (https://itol.embl.de/).

#### ADMIXTURE

For analysis of population structure with the program *ADMIXTURE* [105], the set of strains was filtered for close relatedness using the PGD. The cutoff PGD was chosen to be 0.0007 because lower distances indicated very closely related strains caused by recent selfing events (see Fig 1C). Whenever the PGD was less than or equal to 0.0007, the strain with the largest amount of missing data was removed. The initial sample of *C*. *hirsuta* strains included 25 strains from the Azores to yield a total of 488. Filtering on close relatedness left 358 strains that were used for *ADMIXTURE* analysis. The SNP data were filtered against missing values and correlated SNPs using plink (—geno 0.05—indep-pairwise 30,000 5,000 0.2) leaving 84,065 SNPs. The program *ADMIXTURE* was then run 75 times for each ancestral population (K = 1) through K = 10, with different random seeds. The individual runs were evaluated for lowest cross-validation (CV) error to determine the best K. The result of the individual replicate run with the lowest CV error was used for further analysis. For the analysis of all 753 strains, including all accessions from the Azores, the same procedure was applied yielding a subset of 421 strains and 24,391 SNPs after filtering (—geno 0.05—indep-pairwise 30,000 5,000 0.1). The strains that had been filtered out due to being closely related to retained strains were assigned the same ancestry as the latter.

#### Principal component analysis

PCA of genetic variation data was conducted with the R package *SNPRelate* [106] using all 488 strains. Non-biallelic SNPs were excluded and LD-pruning was applied with a cutoff of 0.2. Strains in the PCA plot were color coded according to the ancestry groups identified in the *ADMIXTURE* analysis.

#### Calculation of nucleotide diversity, Tajima’s D, *F_ST_*, and linkage disequilibrium

Nucleotide diversity, Tajima’s D, and *F*_*ST*_ values in S2 Table were calculated with *vcftools* version 0.1.16 [107]. For *C*. *hirsuta*, nonadmixed accessions from the BAL, NCE, and IBE ancestry groups were used. Exons and low-recombining pericentromeric regions (as defined by [53]) were excluded. Statistics were calculated using a sliding window approach with span and stride of 100 kb. Mean and standard deviation were calculated based on all windows. For *A*. *thaliana*, we created a dataset composed of populations representing counterparts to the *C*. *hirsuta* ancestry groups by relying on VCF files and the *ADMIXTURE* analyses of the 1,001 genome data (1001genomes.org; http://1001genomes.github.io/admixture-map), as well as on the VCF file from [19] for the Madeiran population. Similar criteria as for the *C*. *hirsuta* dataset were used: considering only accessions with less than 5% admixture and filtering out exons and pericentromeric regions (as defined by [108]). Summary statistics were calculated using the same procedure as for *C*. *hirsuta*. LD decay along physical distance of 300 kb was calculated on the same sets of accessions using PopLDdecay [109] after excluding SNPs from pericentromeric regions.

#### *MSMC2* and *relate*

Ancestral changes in coalescence rates among the ancestry groups were determined using the programs *MSMC2* [50] and *relate* [47]. For these and further analyses, 2 additional masks were prepared and combined with those described above: (i) all exons were masked; and (ii) the low-recombining pericentromeric regions were masked. For *MSMC2*, input data were prepared using the masks to determine the total number of observed monomorphic sites between SNPs. Twenty unique datasets were prepared each for the comparisons BAL-IBE, BAL-NCE, and IBE-AZ. Each dataset contained 4 strains per group selected at random from those that showed no evidence of admixture in the *ADMIXTURE* analysis. The selections of 4 strains per group were ensured to be unique. All SNPs for which at least 2 of the 8 strains in a dataset were polymorphic were included while allowing for missing values. The total number of observed sites between the SNPs was counted as the number of sites for which all 8 strains were unmasked. *MSMC2* was then run with the time pattern “1*2+40*1+1*2+1*3” on the within ancestry group strains and across ancestry groups separately. N_e_ was estimated from within group coalescence rates, and the scaled time segments were converted to generations according to the general *MSMC* guide (https://github.com/stschiff/msmc/blob/master/guide.md), using a mutation rate per generation, per nucleotide, of either 4 × 10^−9^ (Figs 1E and 3D) or, for reasons of comparability to earlier works, 7 × 10^−9^ (S1G Fig). Relative cross coalescence rates (RCCRs) were calculated as the sum of both within group coalescence rates divided by twice the across group coalescence rates. When RCCR dropped below 0.5, it was considered the time at which the populations split.

The program *relate* required the SNPs without missing values. In order to prevent the loss of too many SNPs due to this reason, the missing values were imputed using *SHAPIT4* [110] version 4.1.2. with the options “*region 1—thread 20—effective-size 150000—pbwt-depth 10*” and with the genetic map described below. All analyses were performed with *relate* version 1.1.14. Input data were prepared from a VCF file using the module *RelateFileFormats*. Due to the high level of self-fertilization in *C*. *hirsuta*, we considered that each strain contributed a single haploid genome and modified the input files accordingly. Ancestral recombination graphs were constructed for samples of 20 accessions from each of the 4 ancestry groups identified by *ADMIXTURE* analysis as showing no evidence of admixture. Furthermore, low-recombining pericentromeric as well as exonic regions were masked to minimize the effect of direct and indirect selection on the estimations. *relate* was executed using our recombination map and the following options “—mode All -m 4e-9 -N 100000—threads 20”; the analysis was repeated with 2 recently published mutation rates for *A*. *thaliana*: 4·10^−9^ [111] and 7·10^−9^ [17,112]. The tree sequences generated by *relate* for each chromosome were used as input to estimate changes in coalescent rates using the *relate* module *EstimatePopulationSize*.*sh*.

#### Fastsimcoal2

For the demographic analyses using the software *fastsimcoal2* (version 2.6.0.3), we used 20 individuals from each of the 3 ancestry groups identified by the software *ADMIXTURE* that did not show admixture from other groups. Low-recombining pericentromeric as well as exonic regions were filtered out to minimize the effect of direct and indirect negative selection on the site frequency spectrum (SFS). Sites with missing data were excluded, and the number of monomorphic sites (i.e., class 0,0,0 of the observed SFS) was determined by applying the same quality criterion to polymorphic and monomorphic sites. The unfolded SFS for the BAL, NCE, and IBE ancestry groups was constructed using an in-house python script based on the *pyVCF* and *dadi* library [113]; sites with more than 2 alleles were removed. All 5 models considered in this analysis had 3 populations, corresponding to the 3 ancestry groups BAL, NCE, and IBE, and they shared the same population tree topology ((BAL, NCE) IBE) suggested by the *F*_ST_, *PCA*, *MSMC2*, and *relate* analyses. Asymmetric migration rates were allowed to vary freely between all 3 pairs of populations and were constant through time. Sizes for each population were constant except for possible bottlenecks (depending on the model) and a final change in size that was allowed to occur concomitantly with the last population split. A detailed description of the models and a table summarizing all parameters including the range used to pick initial parameter values for likelihood optimization are available in S3 Table. The software *fastsimcoal2* was run with the following options *“-m -n 100000 -M 0*.*01 -N 100000 -l 10 -L 40 -q -x–multiSFS*”; the maximum likelihood for each model was chosen as the largest likelihood among 50 independent runs. Model choice was conducted based on rAIC values, as described in [51]. Two different published mutation rates were used to rescale our estimates into effective population sizes: 4·10^−9^ [111] and 7·10^−9^ [17], and we assumed 1 generation per year to scale the age of demographic events into years. Confidence intervals were obtained by simulating 100 datasets under our best demographic model and reestimating parameter values for each simulated dataset using the same procedure as for the observed data.

### Linking genetic variation to phenotypes in a GWA panel

#### Phenotyping and GWAS

An experiment was conducted in the greenhouse under long-day conditions in which leaflet numbers on the first 10 rosette leaves, and flowering time were quantified on up to 4 and up to 3 replicates, respectively, for each of 352 *C*. *hirsuta* strains. The mean flowering time was used for further analysis. Heteroblasty causes the numbers of leaflets on the rosette leaves to be correlated, and we previously described a negative relationship between the rate of heteroblastic progression and flowering time [25]. The heterchronic effects on leaflet number became apparent particularly on later leaves (>leaf 5), but due to early flowering, some plants did not produce more than 6 leaves. Therefore, rather than using the mean leaflet numbers per leaf node, we fitted an exponential model to these data and used the predicted values at each leaf node as an estimate of the mean. The advantages of this approach included the following: (i) a better estimate of leaflet number per leaf by using information from all leaves; (ii) improved normality due to estimation on a continuous scale rather than ordinal; and (iii) extrapolation of leaflet number up to leaf 10 for plants that did not produce that many leaves due to early flowering. Nevertheless, for GWAS of leaflet number on leaf 7, shown in Fig 3A and 3B, only 5 values were estimated by extrapolation. Quantile normalization was applied to the mean flowering time data to improve normality. Before calculating the kinship matrix for correction of population structure during GWAS, the SNP data were filtered down to 369,241 SNPs as follows using *PLINK2* (www.cog-genomics.org/plink/2.0/): (i) a maximum missing call rate of 0.35; (ii) a minimum minor allele count of 10; (iii) (peri)centromeres excluded; and (iv) a squared correlation (r^2^) less than 0.8. A centered kinship matrix was then calculated using *GEMMA* [114], which included 203,701 of the 369,241 SNPs. GWAS was performed using *GEMMA* with 2,651,721 SNPs that remained after filtering for maximum missing rate of 0.3 and minor allele frequency of 0.04. We also performed GWAS of flowering time using a multilocus mixed model (MLMM) approach [115] as implemented in the R package *mlmm*.*gwas* version 1.0.6. Based on the results of MLMM, we performed a GWAS with the most significant SNP as a covariate using *GEMMA* and used the result to produce Fig 2B.

### Positive selection of a *FRIGIDA* loss-of-function mutation highlights similarities and differences in adaptation to Central European climate in *C*. *hirsuta* and *A*. *thaliana*

#### Chromosome 8 reassembly

As a quality control for the correspondence between the genetic and physical maps of *C*. *hirsuta*, we reconstructed a genetic map from the Ox × Az1 RIL-population from low coverage resequencing data (see Materials and methods paragraph “QTL mapping in the Ox × Az1 RIL population”). The recombination fraction between the genetic markers identified 2 groups of makers assigned on chromosome 8 of the physical map that showed no linkage to other markers of the same chromosome but to chromosome 7 (S9 Table and S6A Fig). Furthermore, the analysis of the genetic map suggested a large segment with inverted order located in the border of the pericentromeric region of chromosome 8 physical map (S6B Fig). The recombination positions of the low coverage resequencing data of the Ox × Az1 RIL population together with the Nanopore reads aligned to the *C*. *hirsuta* genome enabled the identification of the borders of the misassembled regions on the physical map (S9 Table and S6C Fig). To do so, the Nanopore reads were first aligned to the reference genome using *minimap2* with the options (-ax map-ont) [116]. Secondly, in the proximity of the borders of predicted misassembled regions, the reads were inspected for consistent truncations in the alignment that allowed to pinpoint the position of the genome misassembly. After switching to the reassembled genome, Nanopore reads confirmed the new assemblies (S6D and S6E Fig). This assembly was applied to the variant files that were used in the downstream analyses.

#### *FRI* allele prediction

Genetic variants occurring in the *FRIGIDA* gene of 746 *C*. *hirsuta* and 1,212 *A*. *thaliana* strains were filtered from SDI files. Genetic variants of *A*. *thaliana* strains were called from publicly available resequencing data using the IMR-DENOM-IRISAS pipeline [103] to ensure comparability. The sequences of the different *FRIGIDA* alleles were obtained by modifying the reference sequence according to the variants called inside the gene using a custom R script. They were then translated into amino acid sequences in silico using the R package *seqinr* [117]. Truncated alleles were identified and labeled according to the position of the premature stop codon. Alleles with mutations in splicing sites, start codon, or that were 10% shorter than the full-length allele were classified as truncated alleles (S4 Table). For *A*. *thaliana*, it was possible to recover 29 truncated *FRI* alleles including the *FRI*_*col*_ and *FRI*_*Ler*_ alleles. The identified alleles of *A*. *thaliana* agreed in 757 out of 761 cases for full-length and 239 out of 241 cases for truncated *FRI* alleles with predictions from Zhang and Jiménez-Gómez [118], indicating the reliability of this method.

#### *FRIGIDA* constructs, plasmids, and complementation

To obtain *pUBQ10*::*FRIstop1-3* and *pUBQ10*::*FRIfunc*, the *ATUBQ10*::promoter was amplified with the primers TGCAGTACCCGACGAGTCAGTAATAA and CTCGAGAGTGTTAATCAGAAAAACTCA, thereby attaching the 3′ *Pst*I and 5′ *Xho*I restriction sites. This fragment was subcloned into *pJet* 1.2 (Thermo Fisher Scientific) according to the protocol and subsequently cloned as a *Pst*I and *Xho*I fragment into *pBJ36*. The open reading frame of *FRI* was amplified from *C*. *hirsuta* Oxford (*FRIstop1*), *C*. *hirsuta* Japan (*FRIstop2*), *C*. *hirsuta* 858B (*FRIstop3*), and *C*. *hirsuta* Azores1 (*FRIfunc*) using the primers GAATTCATGCAGGAGGAGCAACCCTCAACGGC and GGATCCTTACGTTCTTCCTTTGCGGTCTAGTG, which added 3′ *EcoR*I and 5′ *BamH*I restriction sites. The blunt-ended PCR fragments were subcloned into *pJet1*.*2*. The *FRI* alleles were inserted into *pBJ36-pUBQ10* using *EcoR*I/*BamH*I double digest with subsequent ligation. The *pUBQ10*::*FRI* fragments were then cloned into *pML-BART* using *Not*I. All plasmids were verified by dideoxy-sequencing and transformed in *Agrobacterium tumefaciens* strain *GV3101*. Plants were transformed with *A*. *tumefaciens* by floral dip technique [119]. T1 seeds were collected and sown and the germinated seedlings were treated with BASTA to identify transformed T1 individuals by being Basta resistant. In the experiment, we measured RLN in 20, 41, 48, 49, and 58 plants transformed with *pML-BART*, *FRIfunc*, *FRIstop*, *FRIstop2*, and *FRIstop3*, respectively. We tested for statistical differences between groups using the Dunn test with Bonferroni adjustment for multiple comparisons. Five T2 plants of 20 independent T1 plants of *pUBQ10*::*FRIfunc* and *pUBQ10*::*FRIstop*, respectively, were sown together with 48 control plants derived from 3 independent transgenics transformed with the *pML-BART*. After confirming the presence of the transgene via genotyping for the presence of the BASTA resistance gene using the primers GAAGTCCAGCTGCCAGAAAC and TACATCGAGACAAGCACGGT, we scored leaflet number until leaf 7 on at least 33, 37, and 46 plants of *FRIfunc*, *FRIstop*, and Control, respectively. We tested for statistical differences between all groups on each leaf separately using the Dunn test with Bonferroni adjustment for multiple comparisons.

#### Nucleotide diversity and Tajima’s D in the Northern Central European population

PLINK PED files were converted into VCF files keeping only strains with more than 99% of NCE ancestry proportions using *PLINK1*.*9* [120]. The final dataset contained 12 strains carrying the full-length (*FRIfunc*) and 45 strains carrying the truncated *FRI* allele (*FRIstop*). Pi and Tajima’s D were calculated using *VCFtools* [107] with a window size of 200 kb and a step size of 50 kb.

#### Selective sweep analysis with *SweepFinder2*

We aligned Illumina short reads of the outgroup species *C*. *oligosperma* as previously described and called SNPs at known polymorphic sites using *samtools mpileup*. We retained only SNPs that were polymorphic in NCE strains with more than 99% of NCE ancestry proportions. We polarized these SNPs into ancestral and derived alleles according to the outgroup allele and created a site frequency (SF) file using a custom R script. *SweepFinder2* [59] was run on each chromosome and each group (*FRIstop* and *FRIfunc*) separately, including monomorphic sites at a step size of 10,000 using the precomputed empirical genome-wide frequency spectrum. To obtain a significance threshold for the composite likelihood ratio, 1,000 neutral simulations of chromosome 8 were performed using *msprime* version 0.7.3 [121] based on estimates from the best *fastsimcoal* model (S1I Fig and S3 Table). Recombination rates outside (6.17 × 10^−9^ recombinations per base pair per generation) and inside (4.39 × 10^−10^) pericentromeric regions were estimated from the genetic map described in section 3.1 by regressing genetic distances on physical distances, and a mutation rate of 4 × 10^−9^ mutations per base pair per generation was used. *SweeD* [122] analysis was then performed on all simulated datasets, and the threshold of significance was calculated as the 95th percentile of the maximum CLR values of each simulation. The strongest and significant CLR peak on chromosome 8 ranged from 8,365,935 to 11,617,060 bp. We visualized the SFS, which was U-shaped inside and L-shaped outside of the peak.

#### Simulations of sweep size

Simulations were used to determine if the observed size of the swept region containing *FRIstop* on chromosome 8 was consistent with the relatively recent differentiation of NCE. We used *SLiM* version 3.3 to simulate tree sequences under the Wright–Fisher model [123,124] according to the best demographic model found with *fastsimcoal2* (S1I Fig and S3 Table). A burn-in period of 100 generations forward in time was applied and finalized through recapitation. A chromosome with the length of chromosome 8 in *C*. *hirsuta* was simulated (18,747,412 bp). Recombination rates in the chromosome arms were 9.944·10^−8^ recombinations·bp^−1^·generation^−1^ (rec·bp^−1^·gen^−1^), and a reduced rate of 4.945·10^−9^ rec·bp^−1^·gen^−1^ in the pericentromeric region (according to [53]). The self-fertilization rate was set to 95% per generation.

A selected recessive mutation (h = 0) representing *FRIstop* was introduced in the position 11,100,000, the approximate position of *FRIstop*, at or after NCE split from its ancestral population (BAL) 16,030 generations before present. The simulated tree sequences were overlaid with neutral diversity using *msprime* version 0.4.7 [121] with a mutation rate of 4·10^−9^ mutations·bp^−1^·generation^−1^. All combinations of the selection coefficients 0.001, 0.01, and 0.1 and appearance of *FRIstop* at 16,030, 13,030 10,030, 7,030, 4,030, 1,030 before present were simulated 12 times, except for 1 combination 8 times. Simulations in which *FRIstop* did not achieve the empirical frequency of 79.31% in our *C*. *hirsuta* sample were discarded.

A sliding window analysis for nucleotide diversity (Pi) was performed on the simulated data using *VCFtools* version 0.1.16 [107] with a window size of 200 kb and a stride of 25 kb. The size of the simulated sweep was measured as the region of reduced Pi containing the selected mutation. The boundaries of the sweep were determined by the windows in which Pi was lower than the average Pi of the region 18,000,000 to 18,747,412 bp on the simulated chromosome, which was chosen for being most distant and therefore most weakly linked to the simulated mutation. The simulated sweep size was compared to the empirical sweep size, which was determined to be 6,832,662 bp by piece-wise linear regression of Pi on position.

### The SPL9 transcription factor underlies potentially adaptive heterochronic variation in the Azores

#### *C*. *hirsuta* collection from the Azores

The Az1 strain was collected from the Azorean island of Faial [20]. Afterwards, 4 sampling trips were undertaken, 3 times in late spring, and once in the fall. The number of collected strains that were sequenced from each of the islands per sampling can be found in S10 Table.

Seeds or ripe siliques were collected from multiple individual plants in each visited population, but only 1 plant per population was sequenced, with the exception of 12 individuals from a single population collected from Flores in the fall trip. During the same sampling trip, phenological data were collected for the populations visited. Plants were in situ scored for their developmental stage according to 6 categories, from 1 (very young seedlings) to 6 (mature plants shattering seeds). These data were recorded in several representative patches of each population. At the same time, leaf material was harvested and desiccated using silica gel. DNA extracted from this material was later genotyped for the Az1 allele of *SPL9* in the lab.

#### Phenotyping of Azorean strains

Four replicates each of 264 *C*. *hirsuta* strains collected from the Azores were cultivated in a climatic chamber (Controlled Environments) under long-day conditions. Leaflet numbers were counted on the first 6 rosette leaves. Strains with low leaflet numbers were identified as belonging to the first mode of the leaflet number distribution, which included the Az1 strain.

#### Field experiment on the Azores

Common garden experiments were conducted at the Jardim Botânico do Faial, Azores (Portugal). The seeds were planted on November 13, 2018. Leaflet numbers of 22 Ox wt strains and 19 introgression lines (IL LLN4_2Az1) that contain a single Az1 introgression in the Ox genetic background flanking the *SPL9* locus (see Materials and methods section on Construction and validation of introgression lines) were counted approximately at flowering time of the plants.

#### Leaf silhouettes

Leaf silhouettes were obtained by scanning the leaves, after gluing them to paper, at 800 dpi and a bit depth of 24 with an Epson Perfection V700 Photo scanner. For Fig 3B, the leaves were first converted to black and white silhouettes using Adobe Photoshop and then converted to a vectorized path object using Adobe Illustrator’s Image trace option.

#### Construction of the Ox × Az1 RIL population and its genetic map

The Az1 strain was propagated through single seed descent (SSD) for 3 generations to reduce the amount of potential heterozygosity before it was crossed to the Ox strain. In total, 192 F2 lines were propagated to the F9 generation by SSD. Genetic markers were obtained by Illumina resequencing at a coverage of 0.5× (Max Planck Genome Center, Cologne), and subsequent imputation using *Reconstruction* [125]. One genetic marker was created for each recombination breakpoint. The construction of the genetic map with these markers was performed using a combination of *R/qtl* [126] and *MSTMap* [127]. Duplicated markers, markers with more than 6.25% missing data and markers that departed significantly from a 1:1 segregation ratio (Bonferroni-adjusted *P* value < 0.05), were removed. In parallel, we created a genetic map de novo using *MSTMap* to include markers from unanchored scaffolds using a grouping logarithm of the odds (LOD) of 3, and the Kosambi function. Markers from unanchored scaffolds that could not be mapped to any of the major linkage groups were discarded. In case that adjacent markers of unanchored scaffolds showed a distance larger than 8 cM, these markers were also discarded. The de novo map showed nearly the same order of markers than the physical map, but not entirely. To locate genetically unanchored scaffolds, a marker order was forced according to the physical map of the 8 chromosomes. Markers from unanchored scaffolds were then added to locate them unambiguously based on recombination frequency. As described above, some genetic markers that were originally found on chromosome 8 were transferred to chromosome 7, and the order of markers in the pericentromeric region was inverted. Gaps greater than 5 cM were examined individually to find potential markers of low quality, which would cause an apparent excess of recombination with flanking markers. For that, the length of the genetic map was calculated in the absence and presence of the genetic markers flanking large gaps, and we excluded those markers that caused an increase of 2.5 cM or more. The final map contained 2,720 markers with an average spacing of 0.3 cM, a maximum spacing of 8.9 cM, and a total length of 815.2 cM (see S5 Table).

**Phenotyping of the Ox × Az1 recombinant inbred lines**: Six replicates of the progeny of the resequenced RILs and 12 replicates of the parental lines were sown on moist soil and stratified for 10 days at 4°C. The plants were grown in the greenhouse under long-day conditions. Leaflet numbers on the first 10 rosette leaves and the total number of rosette leaves were scored.

**QTL mapping procedures**: Multitrait QTL analysis was performed using GENSTAT 20th edition [128]. Multitrait QTL mapping scans were performed with the *QMTQTLSCAN* procedure using the mean trait values per genotype for leaflet numbers on leaf nodes 2 to 10 and RLN. A simple interval mapping scan was followed by several rounds of composite interval mapping. During the latter, cofactors were added, removed, or their position was refined until no further improvement of the QTL model could be achieved. The final set of cofactors were then used as genetic predictors in the *QMTESTIMATE* procedure to fit the final QTL model and to estimate the allelic effects and variances explained by those QTL.

The multiple QTL model analysis for each trait separately was performed using *R/qtl* [126] with a custom R script for finding the best QTL model for each trait [129]. New QTL and epistatic interactions were identified using the *SCANTWO* interval mapping function at 1 cM resolution using Haley Knott regression [130]. When no QTL could be identified using *SCANTWO*, new QTL were identified through *SCANONE* interval mapping at 1 cM resolution. All new QTL were added to the model, and the positions were refined using *REFINEQTL*. New QTL were added using *ADDPAIR* and *ADDQTL* and new interactions using *ADDINT*. All models were checked using *FITQTL*, and we retained only QTL above the LOD threshold determined by 10,000 permutations, which corresponds to a LOD of 3.14 for single QTL and 4.17 for epistatic interactions. QTL less than 10 cM apart were considered the same. The search for new QTL was finished when no new QTL could be identified or when the same QTL model occurred twice in the iterative process. The 1.5 LOD interval of each QTL was computed for each QTL in the final QTL model.

#### Selection of heterogeneous inbred families

HIFs are NILs (sister plants) that segregate only for the region of interest in an otherwise homozygous albeit heterogeneous background. HIFs were selected from the F7 generation of the Ox × Az1 RIL population [131]. These were the RIL48 (HIFLLN4_1) and RIL126 (HIFLLN4_2). For the validation of the QTL *LLN4_1*, we selected recombinant lines in the progeny of HIFLLN4_1 using the markers in S11 Table. The progeny of heterozygous HIFs were grown in short-day conditions and genotyped at markers within the segregating region. We phenotyped 17, 18, 19, and 37 plants homozygous for the Az1 allele and 20, 26, 19, and 33 plants homozygous for the Ox allele for HIFs LLN4_1, LLN4_2, LLN4_1A, and LLN4_1B, respectively. We tested for differences in leaflet number between genotypes on each leaf node using the nonparametric pairwise Wilcoxon signed-rank test.

#### Construction and validation of introgression lines

Three ILs were derived carrying Az1 alleles for the *LLN4_1* and *LLN4_2* loci in an Ox genetic background. They were obtained from 2 plants selected from HIF LLN4_1Az1 and HIF LLN4_2Az1, by backcrossing during 4 and 6 generations, respectively, using the *C*. *hirsuta* Ox strain as recurrent parent. Marker-assisted selection with the primers in S12 Table was used to obtain lines with an introgression from Az1 spanning the complete *LLN4* region of chromosome 4 (IL LLN4_1Az1+LLN4_2Az1) and only the *LLN4_2* region. The line spanning the whole region was used to derive ILs with smaller Az1 introgression fragments for the *LLN4_1* locus only.

Lines IL LLN4_1Az1+LLN4_2Az1 and IL LLN4_1Az1 carried introgression fragments from Az1 chromosome 4 of approximately 9.8 Mb and approximately 6.3 Mb, respectively. Both lines had a proximal recombination event on chromosome 4 between markers located at 13.074 Mb and 13.147 Mb. However, line IL LLN4_1 carried the distal recombination point between markers at 17.504 and 19.361 Mb, whereas the introgression of IL LLN4_1Az1+LLN4_2Az1 spanned until the end of chromosome 4 (22.86 Mb). Line IL LLN4_2Az1 was validated by Illumina whole-genome sequencing (Max Planck Genome Centre, Cologne). This also allowed determining the Az1 introgression on chromosome 4 to span from position 20,439,000 bp to 20,891,000 bp (approximately 0.5 Mb) and that the total proportion of the genome derived from Az1 in an Ox background is 3.15%. Seeds derived from heterozygous ILs were grown in short-day conditions and genotyped inside the segregating region with genetic markers listed in S12 Table. We phenotyped 18, 19, and 10 plants homozygous for the Az1 allele and 20, 18, and 10 plants homozygous for the Ox allele for ILs LLN4_1+LLN4_2, LLN4_1, and LLN4_2, respectively.

#### Photoperiod shift experiment

A photoperiod shift experiment was conducted using a HIF that segregated for QTL LLN4_2. Ten plants homozygous for the Ox allele, as well as 10 plants homozygous for the Az1 allele, were shifted from the initial long-day to short-day conditions at 0, 4, 6, 8, 9, 10, 11, 14, 18, 20, and 24 days after sowing. RLN was counted on all plants after they flowered. Statistical analysis was performed in R by fitting a logistic model with RLN as dependent variable and days of long day before shifting as independent variable using the function *nls* with the formula y ~ A / (1 + B * E^−×). The inflection points were considered a measurement for the juvenile-to-adult shift in days of LD. Confidence intervals (95%) of the inflection points were estimated by 10,000 bootstraps, and the differences between the inflection points were considered significant if the confidence intervals did not overlap.

#### Fine-mapping of *SPL9*

Two rounds of fine-mapping were performed from the HIF_LLN4_2, which was segregating on chromosome 4 between 20,280,337 and 21,408,115 bp. The progeny of 8 different recombinant lines was genotyped and phenotyped for leaflet number. The 4 most informative recombinant lines are shown in Fig 4C and 4D. We analyzed 21 and 21, 42 and 32, 21 and 21, and 11 and 8 plants for the recombinant HIFs rec 29, rec37, rec36, and rec39 carrying the Az1 and Ox allele at the segregating region, respectively. For each recombinant HIF, significant differences in leaflet number between the 2 alleles were tested using the Kruskal–Wallis test followed by a Bonferroni adjustment of the resulting *P* values. According to PCR markers, the QTL interval was reduced to 20.570 to 20.619 Mb on chromosome 4 (see S13 Table).

#### CRISPR-Cas9 mutagenesis of *SPL9*

The *spl9* mutant was obtained by CRISPR-Cas9 [132]. A construct containing 2 sgRNA sequences (*SPL9* gRNA1: CCGGGTCAGGCAGAGTCCGG, *SPL9* gRNA2: TCAAACAGACGGGTCCGTGG) were integrated in the *pDE-Cas9* vector optimized for Arabidopsis [133]. *C*. *hirsuta* Ox was transformed and transformants (T1) were selected using BASTA. Sequencing of T2 lines revealed two 1 bp insertions of the nucleotides G and C after positions 53 and 179 relative to the start codon, respectively, leading to a frameshift and a premature stop codon resulting in a peptide of 23 amino acids. The *T-DNA* was segregated out, which was confirmed by PCR and the loss of the BASTA resistance. Homozygous T3 plants were used for genomic complementation experiments. To validate the effect of the *spl9* mutation, 10 replicates of the *spl9* mutant, IL LLN4_2 homozygous for the Az1 allele, and Ox wild type plants were grown in short-day conditions. For each leaf node, leaflet numbers between the 3 different genotypes were compared using Dunn test.

#### Genomic constructs for the analysis of *SPL9*

To obtain the plasmid *pChSPL9*::*gSPL9* with a genomic clone of *SPL9* from Az1 (*gSPL9Az1*) or Ox (*gSPL9Ox*), 6 kb genomic fragments containing the open reading frame (ORF), UTRs, and 3-kb upstream sequences were amplified by PCR from the Ox and Az1 strains using the primers TTTGCGGCCGCGAAGTTAACTCGATCTAAATCAAT and CAGCCGCAGCGAGAGACCAGTTGTTATG. The amplified products were cloned in the *pGEM*-T Easy vector System (Promega). Chimeric constructs with the alleles at the 2 missense SNPs swapped were made from the Az1 and Ox CDS of *SPL9*. For that, the CDSs were digested with *Blp*I, and the resulting fragments were religated, to select for those with the alleles interchanged. *SPL9mixOx_Az1* has the Ox allele at the first SNP in *SPL9* and Az1 at the second SNP and vice versa for the *SPL9mixAz1_Ox*. All the CDS fragments were cloned into *pBJ36* already containing the *ChSPL9Ox* promoter and the terminator octopine synthase (OCS) by digestion with *Kpn*I–*Xma*I. The 2 parental versions of *pChSPL9*::*gSPL9*, as well as the *SPL9mixOx-Az1* and *SPL9mixAz1-Ox*, were transferred to the binary vector *pML-BART* using the *Not*I restriction enzyme. All plasmids were verified by dideoxy-sequencing and transformed in *A*. *tumefaciens* strain *GV3101*. Plants were transformed also with an empty *pML-BART* as control.

We obtained and quantified leaflet number of 12, 19, 32, 12, and 20 T1 plants of HIF LLN4_2 homozygous for the *SPL9Az1* allele transformed with *pML-BART empty*, *gSPL9Az1*, *gSPL9Ox*, *SPL9mixAz1-Ox*, *SPL9mixOx-Az1*, respectively. In the complementation experiment of the *spl9* mutant, we propagated 10 independent T1 lines containing a single copy of the transgene for *gSPL9Az1* and *gSPL9Ox* and 3 for the control line. In the T2 generation, 10 replicates of the complemented *spl9* mutants and 5 replicates of each control line were grown and phenotyped in long-day conditions. Transgene copy number was estimated in the complemented *spl9* mutant and ranged between 0 and 2 copies of the transgene. In the analysis, plants without a transgene were grouped together with the control plants. *Ox* wild-type plants and IL LLN4_2 plants homozygous for the Az1 were grown and phenotyped along with the other lines. Cumulative leaflet number from the first to the eighth leaf was compared between 92, 42, 23, 50, 26, 19, and 15 plants of control, *gSPL9Az1*(1), *gSPL9Az1*(2), *gSPL9Ox*(1), *gSPL9Ox*(2), ILLLN4_22Az1, and Ox, respectively. Statistical differences between groups were tested using the Dunn test using the Bonferroni adjustment method of the *P* values.

#### RNA-seq analyses

For the comparison of the transcriptome of Ox and Az1, seeds were stratified on soil at 4°C for 10 days and then transferred to a growth chamber in short day. Seedlings were cut above the hypocotyl at 7 and 12 days after germination and immediately flash frozen in liquid nitrogen. The lines HIF-LLN4_2 (Rec29) Ox and HIF-LLN4_2 (Rec29) Az1 were grown in long-day conditions. Aboveground tissue of the seedlings was harvested at 12 days after germination. RNA extraction was performed with the Plant Total RNA Kit (Sigma) using the standard protocol. The RNA of 3 replicates of each strain and 2 replicates of each HIF was sequenced in the Max Planck Genome Centre Cologne using Illumina HiSeq3000 and Illumina HiSeq2000, respectively. The Illumina reads were processed as described in [53], resulting in the read counts per gene. These data were analyzed using the R-package *DESeq2* [134]. The count data were normalized using the variance stabilizing transformation, and the *blind* argument was set to FALSE. The *P* value adjustment was performed at a false discovery rate (FDR) of 0.1.

### Alignment of *SPL9* sequences from different Brassicaceae species

For the interspecific comparison of *SPL9* amino acid sequences, 21 different sequences including 2 *SPL9* sequences from the outgroup species *Tarenaya hassleriana* were downloaded from https://phytozome-next.jgi.doe.gov/. The evolutionary history was inferred by using the maximum likelihood method and JTT matrix-based model [135]. The tree with the highest log likelihood (−3,561.01) is shown. Initial trees for the heuristic search were obtained automatically by applying Neighbor-Join and BioNJ algorithms to a matrix of pairwise distances estimated using a JTT model, and then selecting the topology with superior log likelihood value. The tree is drawn to scale, with branch lengths measured in the number of substitutions per site. This analysis involved the 21 sequences in addition to the *SPL9* sequences of *C*. *hirsuta* Ox and *C*. *hirsuta* Az1 amino acid sequences. There was a total of 401 positions in the final dataset. Evolutionary analyses were conducted in MEGA X [136].

### Selective sweep analysis with *pcadapt*

For the analysis with *pcadapt* [78], the SNP data were filtered using *PLINK1*.*9* (https://www.cog-genoimcs.org/plink/) to have a minor allele frequency greater than 0.05 and 20% or less missing data per SNP in the 753 global wild strains including the Azores islands. After filtering, 2,247,751 SNPs remained. Initially, a PCA was performed using *pcadapt* including LD clumping with a threshold of 0.1 to reduce the effect of LD on population structure. The first 9 PCs were above the baseline level of PCs and were retained for the *pcadapt* analysis for outlier detection. The distribution of *P* values calculated from the *pcadapt* analysis was U-shaped, showing an excess of very low and high *P* values. Since a QQ plot showed an excess of deviating *P* values, the calculation of the FDR threshold was inappropriate. Alternatively, we classified the 1,000 SNPs with the lowest *P* values as top outlier SNPs (top 0.00044% of all SNPs). Then, a PCA was performed with the top 1,000 significant SNPs that were located within the *pcadapt* peak and that overlapped with the *SPL9* QTL cluster (located between 14,256,679 bp and 20,661,850 bp on chromosome 4). Two groups of highly diverged strains were defined based on the PCA as follows: grp1 with PC1 < 0 and PC2 > 0.07, grp2 with PC1 > 0.11. Visual inspection of the SNPs in the *SPL9* haplotype using *IGV* [137] allowed the identification of strains with recombinations in this region. PCA of all SNPs, within and outside of the *pcadapt* peak, were performed with *pcadapt* as described above. LD clumping was not applied either for the PCA of the top1,000 SNPs or the PCA of all SNPs within and outside the *pcadapt* peak.

### Calculating nucleotide diversity, Tajima’s D, and *F_ST_*

PLINK ped files were converted into VCF files, keeping only strains belonging to grp1 (*n* = 103) or grp2 (*n* = 50), using *PLINK1*.*9* [120]. Nucleotide diversity (π), Tajima’s D, and weighted *F*_*ST*_ between groups of strains were calculated using *VCFtools* [107] with a window size and stride of 100 kb. Differences in π and Tajima’s D between the genomic background (*n* = 1,766 windows) and the *pcadapt* peak (*n* = 64 windows) were tested using the Kruskal–Wallis test. For plotting the weighted *F*_*ST*_, we kept only windows with more than 700 SNPs to exclude regions with big gaps in the alignment.

### Climate data

Climate data were downloaded from http://worldclim.org/ using a resolution of 30″ [138]. The bioclimatic (BIO) variables for the locations of the strains from the Azorean Islands and Ox were extracted using the R package raster [139]. Additionally, daily data from 8 local weather stations were downloaded using the KNMI Climate Explorer (http://climexp.knmi.nl): PO000008506 (Faial, Azores, Portugal lat 38.52, long −28.63), POM00008501 (Flores, Azores, Portugal, lat 39.455, long −31.131), POM00008512 (Sao Miguel, Azores, lat 37.741, long −25.698), POM00008515 (Santa Maria, Azores, Portugal, lat 36.971, long −25.171), UK000056225 (Oxford, Great Britain, lat 51.77, long −1.27), HRE00105217 (Split_Marjan, Croatia, lat 43.5167, long 16.4331), ITM00016310 (Palinuro, Italy, lat 40.017, long 15.283), and SPE00120566 (Toledo, Spain, lat 39.8844, long −4.0492). For analysis of precipitation, a sliding window was applied on the days of the year with an 11-day window and a stride of 5 days over the time period from 1970 to 2020. In each window, the fraction of days with rain (i.e., more than 0 mm of rain) for the total number of days with observations was calculated. Temperature data were analyzed as mean per day of the year.

### Statistical analysis and data visualizations

Data were analyzed with R (R Core Development Team). Information on the statistical details of the results shown in this manuscript can be found in the respective section of the method details and/or the figure legends. Leaflet number per leaf was compared between different groups (e.g., genotypes) with the nonparametric Wilcoxon-rank test for 2 groups and Kruskal–Wallis test for more than 2 groups. When necessary, *P* values were corrected for multiple testing using either Bonferroni or Benjamini−Hochberg correction. For data preparation and visualization, the R packages *data*.*table*, *KRIS*, *Rmisc*, *packcircles*, *rnaturalearth*, *ggplot2* (with the extensions *scatterpie* and *ggrepel*, *ggforce*, *ggpubr*, *ggnewscale*, and *cowplot*) were used [140–144]. Data were represented as mean ± SEM and/or the individual observations. *P* values < = 0.05 were considered significant. *P* values ≤0.05, ≤0.01, and ≤0.001 were marked with (*), (**), and (***), respectively.

## Supporting information

S1 Fig Population structure and demography of *C*. *hirsuta*. **(A)** CV errors in *ADMIXTURE* analysis with different random seeds. The CV error is plotted against the chosen number of populations (K) for 75 independent analyzes. A lower CV error indicates a better fit to the data. In this analysis, K = 3 was unanimously found to be the best number of populations for the data. (**B**) PCA of SNP data corroborates *ADMIXTURE* analysis. The first PC separates IBE (blue) from the others, while the second PC separates BAL (red) and NCE (yellow). Strains with ancestry in only a single ancestry group in the *ADMIXTURE* analysis (Fig 1A) are shown by darker shades versus lighter shades for admixed strains. (**C**) Hierarcical clustering (hclust) of the PGDs reveals distant relict-like groups. Hclust was used to identify relict-like strains based on PGD, and distant groups among them. The results of hclust are shown as a dendrogram where branch length is a measure of PGD. The first bifurcation separated the strains into 2 groups with high genetic distance between them, one of which predominantly resembled the relict-like group (colored labels) also discovered in Arabidopsis [17] because it contained all IBE strains. In contrast to *ADMIXTURE*, this analysis also allowed the identification of groups represented by small numbers of strains, which led to the discovery of 2 distinct relict-like groups of strains with relatively large genetic distance among them. A group of 11 lineages (magenta) from North West Spain, Sweden, the Netherlands, and New Zealand was identified. In *ADMIXTURE* analysis, these strains were located in either BAL or NCE as admixed lines or they were ungrouped. The large genetic and geographic distance between some of the 11 lineages indicated that they may represent a disparate admixed group, possibly with ancestry of underrepresented groups. (**D**) The 2 relict-like groups discovered with hclust are responsible for the second major mode in the PGD distribution. The PGD distribution of all strains is shown by the black outline. Colors indicate the contribution of the 2 most distant groups of strains identified by hclust of the PGD matrix, and the remaining pairs of strains are shown in gray. In the legend, the PGD groups are assigned to the *ADMIXTURE* clusters (Fig 1A) to which the contained strains belonged either directly or in parentheses. The colors in the distribution indicate the change caused by progressively dropping the respective hclust groups from the data in the order of the legend. The blue area accounts for PGD between strains of the IBE group and all other strains, and the magenta area for PGD between the other relict-like group and all other strains except IBE. (**E**) Relict-like *C*. *hirsuta* strains predominantly resemble the Iberian relicts discovered in Arabidopsis. The geographic distribution of the *C*. *hirsuta* strains is shown with PGD group membership indicated by corresponding colors (S1D Fig). Similar to Iberian relicts in Arabidopsis [17], the *C*. *hirsuta* IBE lines (blue) are found at high frequency on the Iberian Peninsula, but also more broadly in Western Europe and on the Macaronesian islands. Map layers were made with Natural Earth and [142]. (**F**) LD decay in comparable ancestry groups of *C*. *hirsuta* (*C*.*h*.) and *A*. *thaliana* (*A*.*t*.). The average squared correlation (r^2^) between pairs of SNPs is plotted against the average physical distance between them in kilobases. For *A*. *thaliana*, ancestry groups comparable to *C*. *hirsuta* were included: Relicts (*C*.*h*. IBE), NWC_Europe (Western Europe + Central Europe + Germany; *C*.*h*. NCE), IBC (Italy, Balkans, Caucasus; *C*.*h*. BAL), as well as the Iberian nonrelict population (*A*.*t*. Iberia). (**G**, **H**) Piecewise reconstruction of ancestral effective population sizes (Ne) in the 3 *ADMIXTURE* groups using *MSMC2* (**G**) and *relate* (**H**), and estimates of split times between them considering a mutation rate of 7∙10^−9^ mutations per base pair, per generation. The top panel shows ancestral changes in Ne within the *ADMIXTURE* groups plotted against time in years, when considering 1 generation per year. Red, blue, and yellow lines indicate the BAL, IBE, and NCE genetic clusters, respectively. With *MSMC2* (**G**), 20 random sets of 4 strains were analyzed, which are all plotted, while with relate (**H**), all strains were analyzed jointly, hence a single line. The bottom panels show the RCCRs in BAL vs. NCE (solid lines) and IBE vs. BAL (dashed lines). The split times estimated with *fastsimcoal2* (S1I Fig) rescaled for a mutation rate of 7∙10^−9^ are indicated by triangles below the x-axes. Light blue shaded areas in the plots show ancient periods of glaciation according to marine isotope stages 2-4, 6, 8, 10, 12, 14, 16, and 18 [45], respectively, from left to right. The period of the LGM [46] is likewise indicated by the darker blue shade. (**I**) The best demographic model according to *fastsimcoal*2 with a mutation rate of 4∙10^−9^ mutations per generation per base pair, and estimated parameters. The 3 populations identified by *ADMIXTURE* analysis are shown as columns along the y-axis, which indicates time from present at the bottom to more ancient times at the top. The width of the columns is scaled according to the estimated respective Ne, which is also shown in or above the columns themselves. Split times between the populations are shown by arrows connecting the columns, where all lineages from a population merge with its ancestral population. The times at which this occurs are also marked on the y-axis. Bottlenecks are indicated by temporary constrictions but are not to scale. The time at which a bottleneck period starts when looking backwards in time is indicated on the y-axis. Bidirectional arrows below the figure indicate the corresponding modes of migration in the model. See S3 Table for all estimated parameters and their confidence intervals. The data underlying the graphs shown in the figure can be found at https://doi.org/10.5281/zenodo.7907435. BAL, Balkan; CV, cross-validation; hclust, hierarchical clustering; IBE, Iberian; LD, linkage disequilibrium; LGM, last glacial maximum; NCE, Northern Central European; PC, principal component; PCA, principal component analysis; PGD, pairwise genetic distance.
(TIFF)

S2 Fig Selective sweep at *FRIGIDA* in Northern and Central Europe. **(A)** Variation of flowering time in days after germination and lateral leaflet number of the rosette leaves 2 to 9 (L2-L9) within the 3 *ADMIXTURE* ancestry groups of *C*. *hirsuta* (see also Fig 1A and 1B). (**B**) Alignment of *C*. *hirsuta* (CH) *FRIGIDA* and *A*. *thaliana* (AT) *FRIGIDA* amino acid sequences. The high frequency *FRIstop* allele in *C*. *hirsuta* and the resulting truncated protein are indicated in blue. (**C**) The presence of the *FRIstop* allele (black circles) indicated in the PCA shown in S1B Fig. *FRIstop* is not found among the set of 85 and 83 nonadmixed lines from IBE and BAL, respectively, and also only sporadically in admixed lines from those groups. In NCE, the frequency is 45 out of 57 nonadmixed lines after removing closely related strains, where it is primarily found in this group. (**D**) Transgenic lines harboring a functional *FRIGIDA* allele exhibit reduced leaflet number in later leaf nodes compared to transgenic lines harboring the *FRIstop* allele and control plants harboring an empty vector (Dunn test with Bonferroni adjusted *P* value, ***: *P* value < 0.001). Black crosses represent the mean ± SEM. (**E**) Evidence for a selective sweep at the *FRIGIDA* locus. Genome-wide sliding window analyses of nucleotide diversity (PI, top), Tajima’s D (middle) are shown as well as the CLR analysis (bottom). The analyses were performed separately in strains with the *FRIstop* (blue) and the *FRIfunc* (black) alleles from the NCE population (Fig 1A). Note how the region surrounding the *FRI* locus (orange dashed line) displays reduced PI, reduced Tajima’s D, and high CLR, consistent with a selective sweep, exclusively in strains with *FRIstop*. The horizontal dashed lines in the top and middle panels indicate the genome-wide averages for the respective groups in blue or gray, and in the lower panel, the horizontal dashed line indicates the threshold (α = 0.05) derived from neutral simulations. (**F**) Graphical representation of allele frequencies on chromosome 8 reveal extended haplotype blocks containing the *FRI* locus (dashed orange line). Major (black) and minor (white) alleles for all SNPs with a minor allele frequency greater than 5% are shown for the NCE ancestry group. Note the reduced genetic diversity around the *FRI* locus in *FRIstop* harboring strains (bottom) as compared to those with *FRIfunc*. (**G**) SFS of SNPs in *FRIstop* harboring strains from the NCE group. The SFS outside of the selective sweep region (Fig 2G) is shown in black and that from within the selective sweep region in orange. (**H**) Physical sizes of swept regions as a function of selection coefficient and duration in simulations indicate that the large haplotype block containing *FRIstop* in NCE is consistent with recent strong selection. Selection on the *FRI* locus was simulated in the context of the empirical local recombination rates estimated for chromosome 8 in *C*. *hirsuta* for the durations indicated on the x-axis. The physical size of the simulated chromosome was identical to the actual size of chromosome 8 and is indicated by the horizontal dotted line. The median sweep sizes from the simulations are shown by the colored lines and their 95% quantiles by the error bars, where red, blue, and yellow indicate simulated selection coefficients of 0.001, 0.01, and 0.1, respectively. The estimated size of the swept region containing *FRI* in our *C*. *hirsuta* sample is shown by the continuous horizontal line. Frequencies of the various truncated *FRI* alleles in *A*. *thaliana* (AT) and *C*. *hirsuta* (CH). Alleles are named as “at” or “ch” followed by the physical position of the polymorphism generating the truncation. Alleles marked with an “x” are found only in strains from outside of Europe. The data underlying the graphs shown in the figure can be found at https://doi.org/10.5281/zenodo.7907435. BAL, Balkan; CLR, composite likelihood ratio; IBE, Iberian; NCE, Northern Central Europe; PCA, principal component analysis; SFS, site frequency spectrum.
(TIFF)

S3 Fig Genetic basis of low leaflet number phenotype in the Azorean *C*. *hirsuta* strain. **(A)** Regression of leaflet number on flowering time in IBE strains (blue) and the remaining strains (gray). Points show the cumulative leaflet number of the first 7 rosette leaves of individual strains, and the lines show the fitted linear models. *P* values and R^2^ of the regressions are shown in the top right corner for both groups. An asterisk (*) indicates the Azores (Az1) strain showing early flowering time and low leaflet number. (**B**) Histogram of cumulative leaflet number on the first 6 rosette leaves of *C*. *hirsuta* strains collected from the Azores. Strains considered to share a low-leaflet phenotype are indicated by the blue shaded area. (**C**) CV errors in *ADMIXTURE* analysis with different random seeds. The CV error is plotted against the chosen number of populations (K) for 75 independent analyzes. A lower CV error indicates a better fit to the data. In this analysis, K = 4 was found to be the best number of populations for the data. (**D**) Piecewise reconstruction of ancestral effective population sizes (Ne) in the 4 *ADMIXTURE* groups (Fig 3C) using *relate*, and estimates of split times between them. The top panel shows ancestral changes in Ne within the *ADMIXTURE* groups plotted against time in years, when considering 1 generation per year. Red, blue, and yellow lines indicate the BAL, IBE, and NCE genetic clusters, respectively. The bottom panels show the RCCRs in BAL vs. NCE (solid lines), IBE vs. BAL (dashed lines), and IBE vs. AZ (dotted line). Light blue shaded areas in the plots show ancient periods of glaciation according to marine isotope stages 2-4, 6, 8, 10, 12, 14, 16, and 18 [45], respectively, from left to right. The period of the LGM [46] is likewise indicated by the darker blue shade. (**E**) Multiple QTL model mapping results for different leaflet number traits and RLN in the Ox × Az1 RIL population. Estimated QTL positions are indicated by the solid black symbols. QTL that are involved in a 2-way epistatic interaction share the same open symbol. The boxes around the QTL represent the 1.5 LOD intervals and indicate the direction of the effect (blue: Ox alleles increase the phenotypic value, red: Az1 alleles increase value) and the percentage of variation explained (color intensity according to the legend). Effects of *LLN4_1* and *LLN4_2* estimated in ILs carrying Az1 alleles at one or both loci. Points show the leaflet number on leaves of individual replicates plotted against leaf nodes. The vertical bars show the standard error of the mean leaflet numbers. Plants homozygous for Ox (Oxford parent strain) or Az1 alleles (ILs) are shown in yellow or blue, respectively. Significant differences according to Kruskal–Wallis tests are shown with asterisks: *: *P* ≤ 0.05; **: *P* ≤ 0.01; ***: *P* ≤ 0.001. The data underlying the graphs shown in the figure can be found at https://doi.org/10.5281/zenodo.7907435. Az1, Azores1; BAL, Balkan; CV, cross-validation; IBE, Iberian; IL, introgression line; LGM, last glacial maximum; NCE, Northern Central European; Ox, Oxford; QTL, quantitative trait locus; RCCR, relative cross coalescence rate; RIL, recombinant inbreeding line; RLN, rosette leaf number.
(TIFF)

S4 Fig Natural variation at *SPL9*. **(A)** Leaflet number progression of *C*. *hirsuta* Ox, the *Chspl9* mutant, and the introgression line IL-LLN4_2, all in the Ox genetic background. Differences in leaflet numbers between the 3 genotypes were tested with a Dunn test and the *P* values, adjusted according to the Bonferroni method, are indicated by asterisks: *: *P* ≤ 0.05; **: P ≤ 0.01; ***: *P* ≤ 0.001. (**B**, **C**) Genome-wide RNA-seq analyses of entire seedlings. (**B**) Comparison of *C*. *hirsuta* Az1 and *C*. *hirsuta* Ox, and (**C**) the NILs HIF-LLN4_2 (Rec29) with Az1 and Ox alleles at the *SPL9* region. Negative log base 10 transformed *P* values are plotted against fold change of expression and each point is a gene. Red-colored points are significantly differentially expressed, while the black ones are not. The *SPL9* gene is indicated in each plot. (**D**) Phylogeny and homology of *SPL9* genes in 16 Brassicaceae. The left panel shows the *SPL9* gene tree. The top panel shows the proportion of genes harboring the most common AA. The bottom-middle panel shows the entire *SPL9* protein sequence, while the bottom-right panel corresponds to the region around the *SPL9* missense SNPE242Q (indicated by asterisk). The data underlying the graphs shown in the figure can be found at https://doi.org/10.5281/zenodo.7907435. AA, amino acid; Az1, Azores1; *Chspl9*, *C*. *hirsuta* loss-of-function allele of *SPL9*; Ox, Oxford; *SPL9*, *SQUAMOSA PROMOTER BINDING PROTEIN-LIKE 9*.
(TIFF)

S5 Fig Genetic divergence at the *SPL9* QTL cluster agrees with climatic gradient on the Azores. **(A)** Reduced leaflet number in an IL carrying the *SPL9Az1* locus in the Ox background (IL LLN4_2Az1) compared to the wild type when grown in a common garden on the island of Faial, Azores. The difference between the means was tested for significance with the Wilcoxon rank-sum test. (**B**) Genome-wide scan for selection with *pcadapt*. A Manhattan plot is shown with the results from the analysis of 753 *C*. *hirsuta* strains. The negative log base 10 transformed *P* values for SNPs are plotted against their physical positions on each chromosome. The dashed horizontal line indicates the genome-wide threshold on or above which there are only 1,000 SNPs. The functional missense SNP E242Q of *SPL9* underlying QTL LLN4_2 is highlighted by a red circle. Yellow boxes in the lower part of the panel indicate the locations of QTL LLN4_1A, LLN4_1B, and LLLN4_2. (**C**) PCA of 753 *C*. *hirsuta* strains with *pcadapt* using all SNPs (minor allele frequency > = 5%) outside the *pcadapt* peaks at the *SPL9* QTL cluster (GBG, left) and inside (*SPL9* cluster, right). Each point is a strain and colors indicate whether it belongs to the Western Azores group grp1 (blue), Eastern Azores group grp2 (red), or others (gray). (**D**) An east–west climatic gradient on the Azorean archipelago as indicated by precipitation of the driest quarter (BIO17) and the distribution of strains grouped according to *pcadapt* analysis (S5A and S5B Fig). Each independent sampling is represented by a pie chart, indicated by season (S, spring; F, fall) and year (e.g., S10 –Spring 2010). Pie charts show the proportions of strains from the different groups in our sample colored according to Fig 5B (blue—grp1; red—grp2; black—recombinant in the *SPL9* cluster; gray—others). The size of the pie chart is scaled to the number of strains according to the legend in the bottom left. Collection locations are indicated on the map by points colored according to BIO17 as indicated by the legend on the right. The latitudinal and longitudinal gradients for BIO17 across the collection sites on the Azores are also indicated along the upper and right margins of the figure. Map layers were made with Natural Earth and [142]. (**E**) Boxplots showing climatic differences between locations of *C*. *hirsuta* strains belonging to grp1 from West Azores (blue) and grp2 from East Azores (red). Only bioclimatic variables that were significantly different between both groups according to a Kruskal–Wallis test are shown. The *P* values of the test are indicated by asterisks: * 0.01 < *P* ≤ 0.05, *** *P* ≤ 0.001. (**F**) Geographic locations of weather stations from which data were analyzed. Stations were chosen to represent the 4 major groups discovered with *ADMIXTURE* analysis (Fig 3C; AZ, IBE, BAL, NCE) based on abundance of strains at their geographical locations. Map layers were made with Natural Earth and [142]. Annual trends in temperature in local weather station data from the AZ and from locations representing the other *ADMIXTURE* groups (S5E Fig). Daily mean temperature is plotted against day of the year. Annual changes in temperature on the Azores are much reduced compared to the other locations primarily due to mild temperature in both winter and summer. The data underlying the graphs shown in the figure can be found at https://doi.org/10.5281/zenodo.7907435. AZ, Azores; BAL, Balkan; GBG, genomic background; IBE, Iberian; IL, introgression line; NCE, Northern Central European; Ox, Oxford; PCA, principal component analysis; QTL, quantitative trait locus; *SPL9*, *SQUAMOSA PROMOTER BINDING PROTEIN-LIKE 9*.
(TIFF)

S6 Fig Reordering of chromosome 8. **(A)** The RF of genetic markers within the Ox × Az1 RIL population reveal 2 regions on chromosome 8 that most likely belong to chromosome 7 instead of previous assignment to chromosome 8 (arrows). In addition, markers of the pericentromeric region (gray diagonal line) might be inverted. (**B**, **C**) Genetic (**B**) and physical (**C**) maps based on previous (left side) and new assembly (right side) of chromosome 8. Regions colored in red, yellow, and blue depict unchanged segments of the map, whereas the region in gray color corresponds to the inverted segment. A genetic map with inverted order of markers shows reduced genetic length supporting the inverted assembly (**B**). The breakpoints of the predicted inversion indicated by 1 and 2 have been previously uncovered by consistent breakpoints in Nanopore long reads mapped to the original assembly of chromosome 8 (**C**). (**D**, **E**) Images representing Nanopore reads (positive strand–blue; negative strand -red) that span the regions 1 (**D**) and 2 (**E**) of the reassembled chromosome 8 and confirm the correctness of the new assembly. The data underlying the graphs shown in the figure can be found at https://doi.org/10.5281/zenodo.7907435. Az1, Azores1; Ox, Oxford; RF, recombination fraction; RIL, Recombinant Inbred Line.
(TIFF)

S1 Table Sample location and Illumina sequencing information of all 752 *C*. *hirsuta* natural strains. (CSV)

S2 Table Population genetic summary statistics in *C*. *hirsuta* and *A*. *thaliana*. (XLSX)

S3 Table Demographic analyses of European *C*. *hirsuta* populations with *fastsimcoal2*. (XLSX)

S4 Table Analysis of *FRIGIDA* alleles in natural strains of *C*. *hirsuta* and *A*. *thaliana*. (CSV)

S5 Table Linkage map of the Ox-Az1-RIL population. (CSV)

S6 Table QTL mapping summary. (XLSX)

S7 Table Collection details of Azorean strains. (CSV)

S8 Table Summary of Illumina read coverage for the 752 resequenced strains. (XLSX)

S9 Table Positions for new assembled regions of the reference chromosome 8. (XLSX)

S10 Table *C*. *hirsuta* strains sequenced from the Azores arranged by island and sampling trip. (XLSX)

S11 Table Primers used for selecting recombinant HIFs. (XLSX)

S12 Table Primers used for developing ILs. (XLSX)

S13 Table Primers used for fine-mapping QTL LLN4_2. (XLSX)

## Abbreviations

AZ: Azores
Az1: Azores1
BAL: Balkan
CDS: Coding sequence
*Chspl9*: *C*. *hirsuta* loss-of-function allele of *SPL9*
CV: cross-validation
FDR: false discovery rate
*FLC*: *FLOWERING LOCUS C*
*FRI*: *FRIGIDA*
GWA: genome-wide association
HIF: heterogeneous inbred family
IBE: Iberian
IL: introgression line
LD: linkage disequilibrium
LLN4: Leaflet number QTL on chromosome 4
MLMM: multilocus mixed model
NCE: Northern Central European
NIL: near-isogenic line
Ox: Oxford
PC: principal component
PCA: principal component analysis
PGD: pairwise genetic distance
QTL: quantitative trait locus
RCCR: relative cross coalescence rate
RIL: recombinant inbreeding line
RLN: rosette leaf number
SF: site frequency
SFS: site frequency spectrum
SNP: single nucleotide polymorphism
*SPL9*: *SQUAMOSA PROMOTER BINDING-LIKE 9*
SSD: single seed descent
TPPI: TREHALOSE-6-PHOSPHATE-PHOSPHATASE I
