# Supplementary figures and images for: Pan-European study of genotypes and phenotypes in the Arabidopsis relative Cardamine hirsuta reveals how adaptation, demography, and development shape diversity patterns
Source: PLoS Biol. 2023 Jul 18;21(7):e3002191. doi: 10.1371/journal.pbio.3002191 (PMC10353826; doi:10.1371/journal.pbio.3002191)

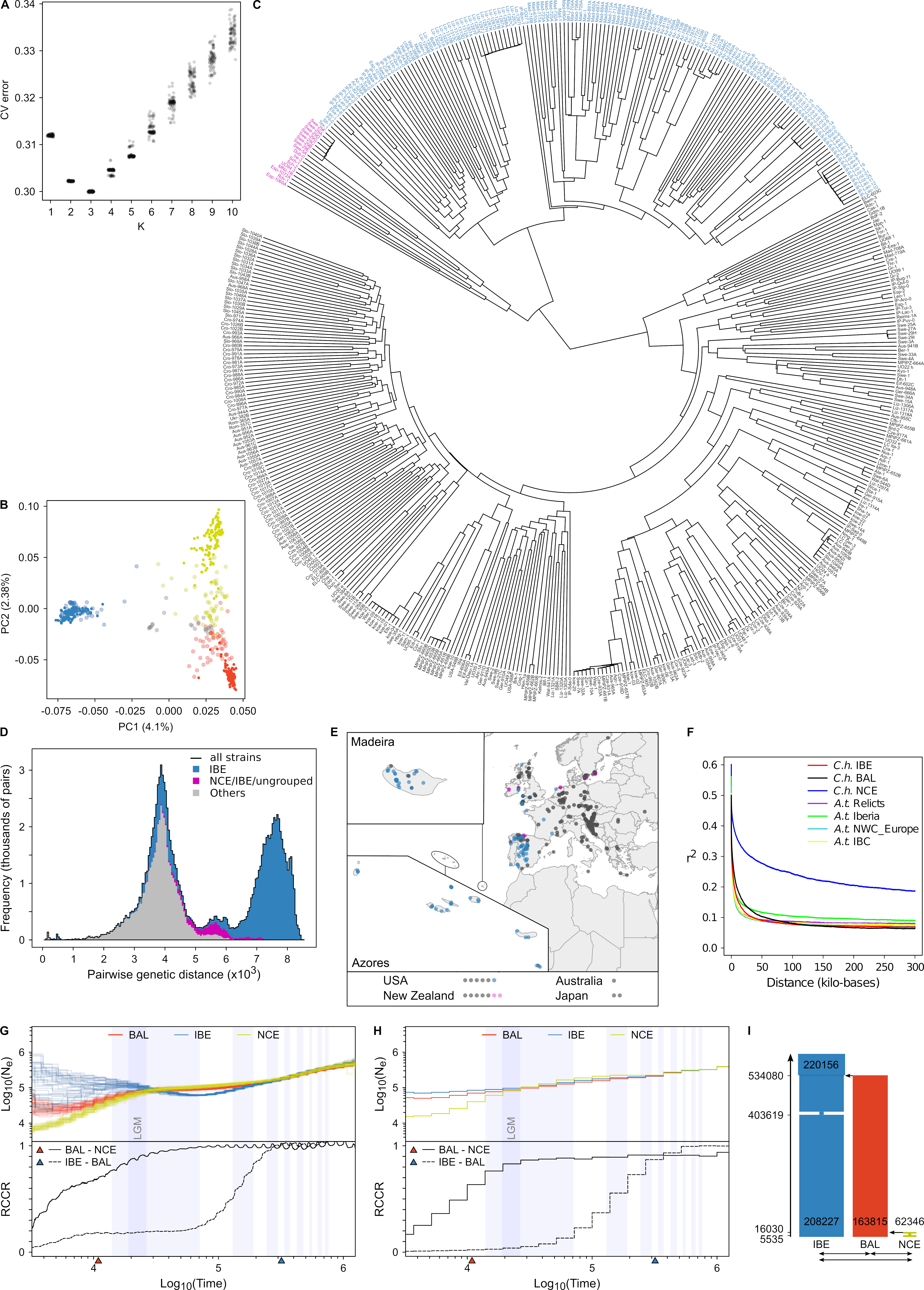

Supplement: S1 Fig — (A) CV errors in ADMIXTURE analysis with different random seeds. The CV error is plotted against the chosen number of populations (K) for 75 independent analyzes. A lower CV error indicates a better fit to the data. In this analysis, K = 3 was unanimously found to be the best number of populations for the data. (B) PCA of SNP data corroborates ADMIXTURE analysis. The first PC separates IBE (blue) from the others, while the second PC separates BAL (red) and NCE (yellow). Strains with ancestry in only a single ancestry group in the ADMIXTURE analysis (Fig 1A) are shown by darker shades versus lighter shades for admixed strains. (C) Hierarcical clustering (hclust) of the PGDs reveals distant relict-like groups. Hclust was used to identify relict-like strains based on PGD, and distant groups among them. The results of hclust are shown as a dendrogram where branch length is a measure of PGD. The first bifurcation separated the strains into 2 groups with high genetic distance between them, one of which predominantly resembled the relict-like group (colored labels) also discovered in Arabidopsis [17] because it contained all IBE strains. In contrast to ADMIXTURE, this analysis also allowed the identification of groups represented by small numbers of strains, which led to the discovery of 2 distinct relict-like groups of strains with relatively large genetic distance among them. A group of 11 lineages (magenta) from North West Spain, Sweden, the Netherlands, and New Zealand was identified. In ADMIXTURE analysis, these strains were located in either BAL or NCE as admixed lines or they were ungrouped. The large genetic and geographic distance between some of the 11 lineages indicated that they may represent a disparate admixed group, possibly with ancestry of underrepresented groups. (D) The 2 relict-like groups discovered with hclust are responsible for the second major mode in the PGD distribution. The PGD distribution of all strains is shown by the black outline. Colors in [file pbio.3002191.s001.tiff]

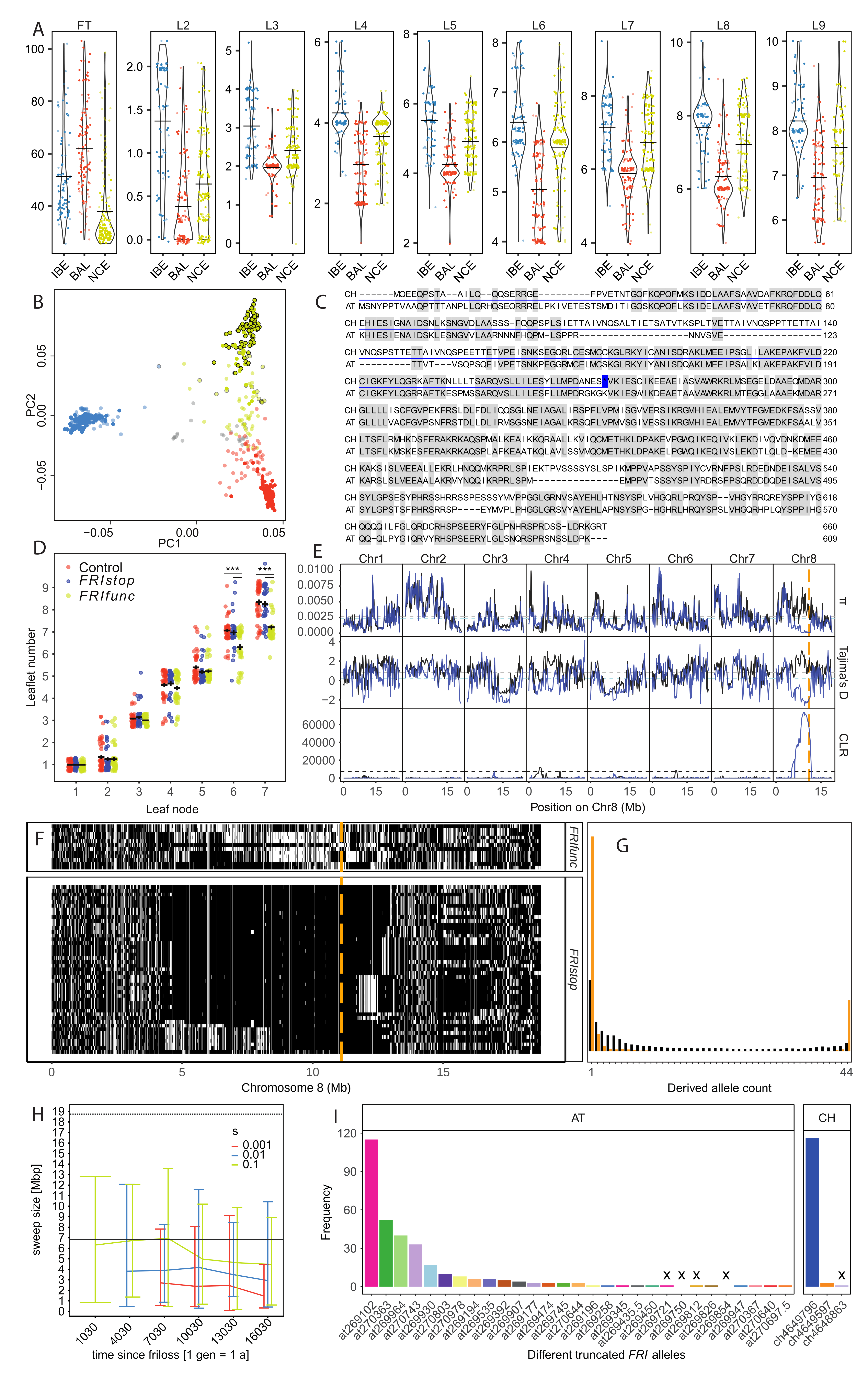

Supplement: S2 Fig — (A) Variation of flowering time in days after germination and lateral leaflet number of the rosette leaves 2 to 9 (L2-L9) within the 3 ADMIXTURE ancestry groups of C. hirsuta (see also Fig 1A and 1B). (B) Alignment of C. hirsuta (CH) FRIGIDA and A. thaliana (AT) FRIGIDA amino acid sequences. The high frequency FRIstop allele in C. hirsuta and the resulting truncated protein are indicated in blue. (C) The presence of the FRIstop allele (black circles) indicated in the PCA shown in S1B Fig. FRIstop is not found among the set of 85 and 83 nonadmixed lines from IBE and BAL, respectively, and also only sporadically in admixed lines from those groups. In NCE, the frequency is 45 out of 57 nonadmixed lines after removing closely related strains, where it is primarily found in this group. (D) Transgenic lines harboring a functional FRIGIDA allele exhibit reduced leaflet number in later leaf nodes compared to transgenic lines harboring the FRIstop allele and control plants harboring an empty vector (Dunn test with Bonferroni adjusted P value, ***: P value < 0.001). Black crosses represent the mean ± SEM. (E) Evidence for a selective sweep at the FRIGIDA locus. Genome-wide sliding window analyses of nucleotide diversity (PI, top), Tajima’s D (middle) are shown as well as the CLR analysis (bottom). The analyses were performed separately in strains with the FRIstop (blue) and the FRIfunc (black) alleles from the NCE population (Fig 1A). Note how the region surrounding the FRI locus (orange dashed line) displays reduced PI, reduced Tajima’s D, and high CLR, consistent with a selective sweep, exclusively in strains with FRIstop. The horizontal dashed lines in the top and middle panels indicate the genome-wide averages for the respective groups in blue or gray, and in the lower panel, the horizontal dashed line indicates the threshold (α = 0.05) derived from neutral simulations. (F) Graphical representation of allele frequencies on chromosome 8 reveal extended haplotype blocks con [file pbio.3002191.s002.tiff]

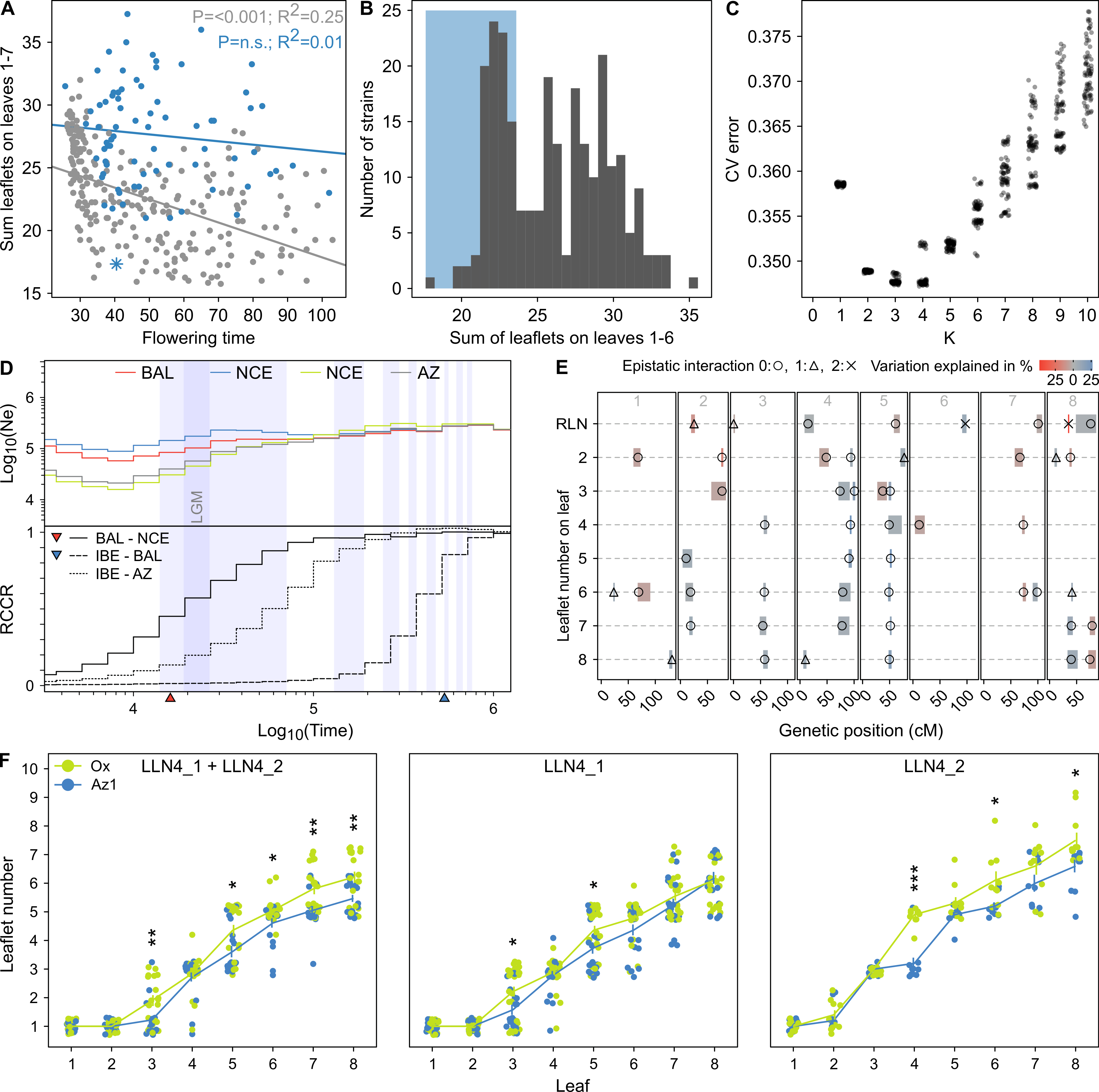

Supplement: S3 Fig — (A) Regression of leaflet number on flowering time in IBE strains (blue) and the remaining strains (gray). Points show the cumulative leaflet number of the first 7 rosette leaves of individual strains, and the lines show the fitted linear models. P values and R2 of the regressions are shown in the top right corner for both groups. An asterisk (*) indicates the Azores (Az1) strain showing early flowering time and low leaflet number. (B) Histogram of cumulative leaflet number on the first 6 rosette leaves of C. hirsuta strains collected from the Azores. Strains considered to share a low-leaflet phenotype are indicated by the blue shaded area. (C) CV errors in ADMIXTURE analysis with different random seeds. The CV error is plotted against the chosen number of populations (K) for 75 independent analyzes. A lower CV error indicates a better fit to the data. In this analysis, K = 4 was found to be the best number of populations for the data. (D) Piecewise reconstruction of ancestral effective population sizes (Ne) in the 4 ADMIXTURE groups (Fig 3C) using relate, and estimates of split times between them. The top panel shows ancestral changes in Ne within the ADMIXTURE groups plotted against time in years, when considering 1 generation per year. Red, blue, and yellow lines indicate the BAL, IBE, and NCE genetic clusters, respectively. The bottom panels show the RCCRs in BAL vs. NCE (solid lines), IBE vs. BAL (dashed lines), and IBE vs. AZ (dotted line). Light blue shaded areas in the plots show ancient periods of glaciation according to marine isotope stages 2-4, 6, 8, 10, 12, 14, 16, and 18 [45], respectively, from left to right. The period of the LGM [46] is likewise indicated by the darker blue shade. (E) Multiple QTL model mapping results for different leaflet number traits and RLN in the Ox × Az1 RIL population. Estimated QTL positions are indicated by the solid black symbols. QTL that are involved in a 2-way epistatic interaction share the same open symbol. The boxes [file pbio.3002191.s003.tiff]

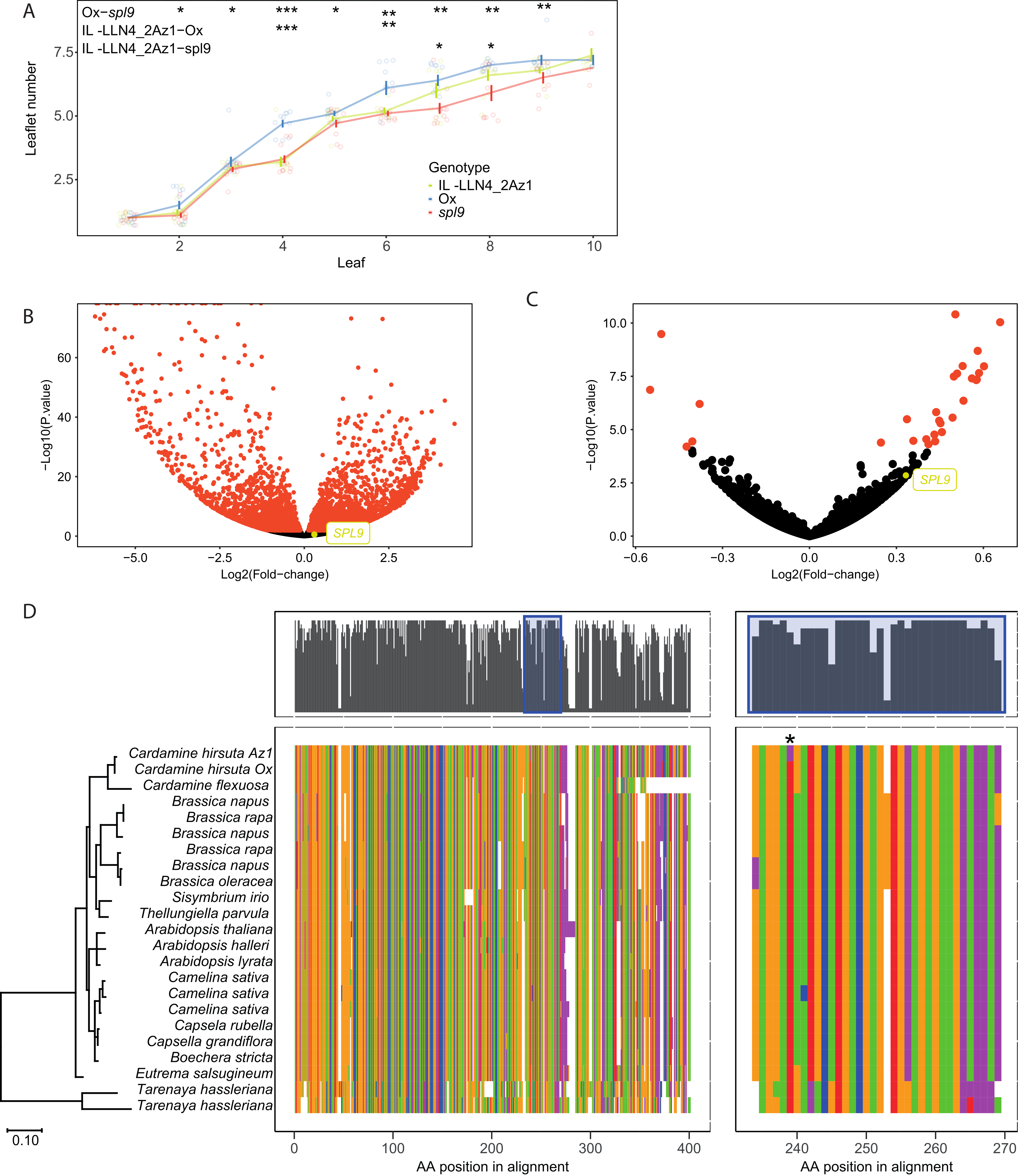

Supplement: S4 Fig — (A) Leaflet number progression of C. hirsuta Ox, the Chspl9 mutant, and the introgression line IL-LLN4_2, all in the Ox genetic background. Differences in leaflet numbers between the 3 genotypes were tested with a Dunn test and the P values, adjusted according to the Bonferroni method, are indicated by asterisks: *: P ≤ 0.05; **: P ≤ 0.01; ***: P ≤ 0.001. (B, C) Genome-wide RNA-seq analyses of entire seedlings. (B) Comparison of C. hirsuta Az1 and C. hirsuta Ox, and (C) the NILs HIF-LLN4_2 (Rec29) with Az1 and Ox alleles at the SPL9 region. Negative log base 10 transformed P values are plotted against fold change of expression and each point is a gene. Red-colored points are significantly differentially expressed, while the black ones are not. The SPL9 gene is indicated in each plot. (D) Phylogeny and homology of SPL9 genes in 16 Brassicaceae. The left panel shows the SPL9 gene tree. The top panel shows the proportion of genes harboring the most common AA. The bottom-middle panel shows the entire SPL9 protein sequence, while the bottom-right panel corresponds to the region around the SPL9 missense SNPE242Q (indicated by asterisk). The data underlying the graphs shown in the figure can be found at https://doi.org/10.5281/zenodo.7907435. AA, amino acid; Az1, Azores1; Chspl9, C. hirsuta loss-of-function allele of SPL9; Ox, Oxford; SPL9, SQUAMOSA PROMOTER BINDING PROTEIN-LIKE 9. (TIFF) [file pbio.3002191.s004.tiff]

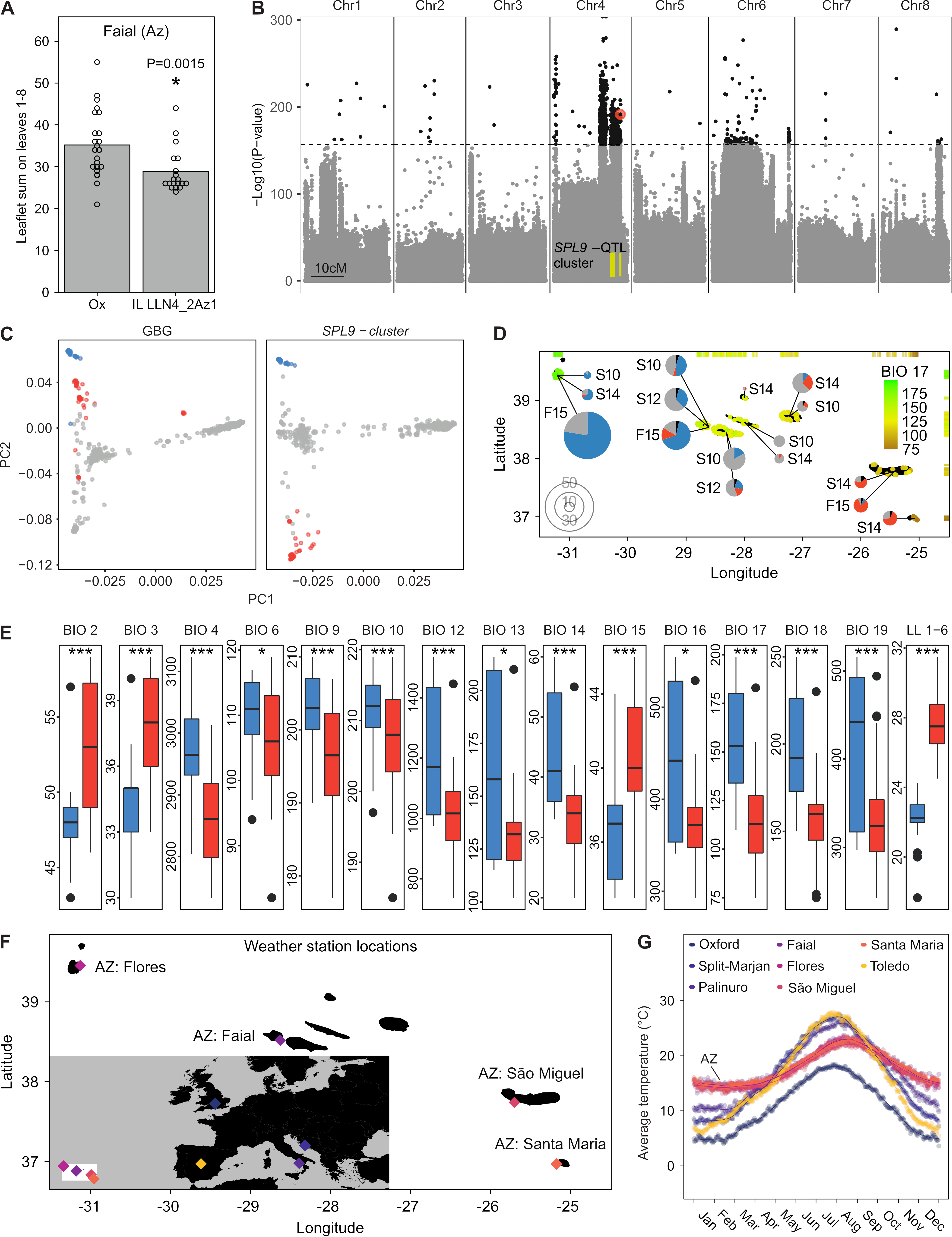

Supplement: S5 Fig — (A) Reduced leaflet number in an IL carrying the SPL9Az1 locus in the Ox background (IL LLN4_2Az1) compared to the wild type when grown in a common garden on the island of Faial, Azores. The difference between the means was tested for significance with the Wilcoxon rank-sum test. (B) Genome-wide scan for selection with pcadapt. A Manhattan plot is shown with the results from the analysis of 753 C. hirsuta strains. The negative log base 10 transformed P values for SNPs are plotted against their physical positions on each chromosome. The dashed horizontal line indicates the genome-wide threshold on or above which there are only 1,000 SNPs. The functional missense SNP E242Q of SPL9 underlying QTL LLN4_2 is highlighted by a red circle. Yellow boxes in the lower part of the panel indicate the locations of QTL LLN4_1A, LLN4_1B, and LLLN4_2. (C) PCA of 753 C. hirsuta strains with pcadapt using all SNPs (minor allele frequency > = 5%) outside the pcadapt peaks at the SPL9 QTL cluster (GBG, left) and inside (SPL9 cluster, right). Each point is a strain and colors indicate whether it belongs to the Western Azores group grp1 (blue), Eastern Azores group grp2 (red), or others (gray). (D) An east–west climatic gradient on the Azorean archipelago as indicated by precipitation of the driest quarter (BIO17) and the distribution of strains grouped according to pcadapt analysis (S5A and S5B Fig). Each independent sampling is represented by a pie chart, indicated by season (S, spring; F, fall) and year (e.g., S10 –Spring 2010). Pie charts show the proportions of strains from the different groups in our sample colored according to Fig 5B (blue—grp1; red—grp2; black—recombinant in the SPL9 cluster; gray—others). The size of the pie chart is scaled to the number of strains according to the legend in the bottom left. Collection locations are indicated on the map by points colored according to BIO17 as indicated by the legend on the right. The latitudinal and longitudinal gradients for BIO [file pbio.3002191.s005.tiff]

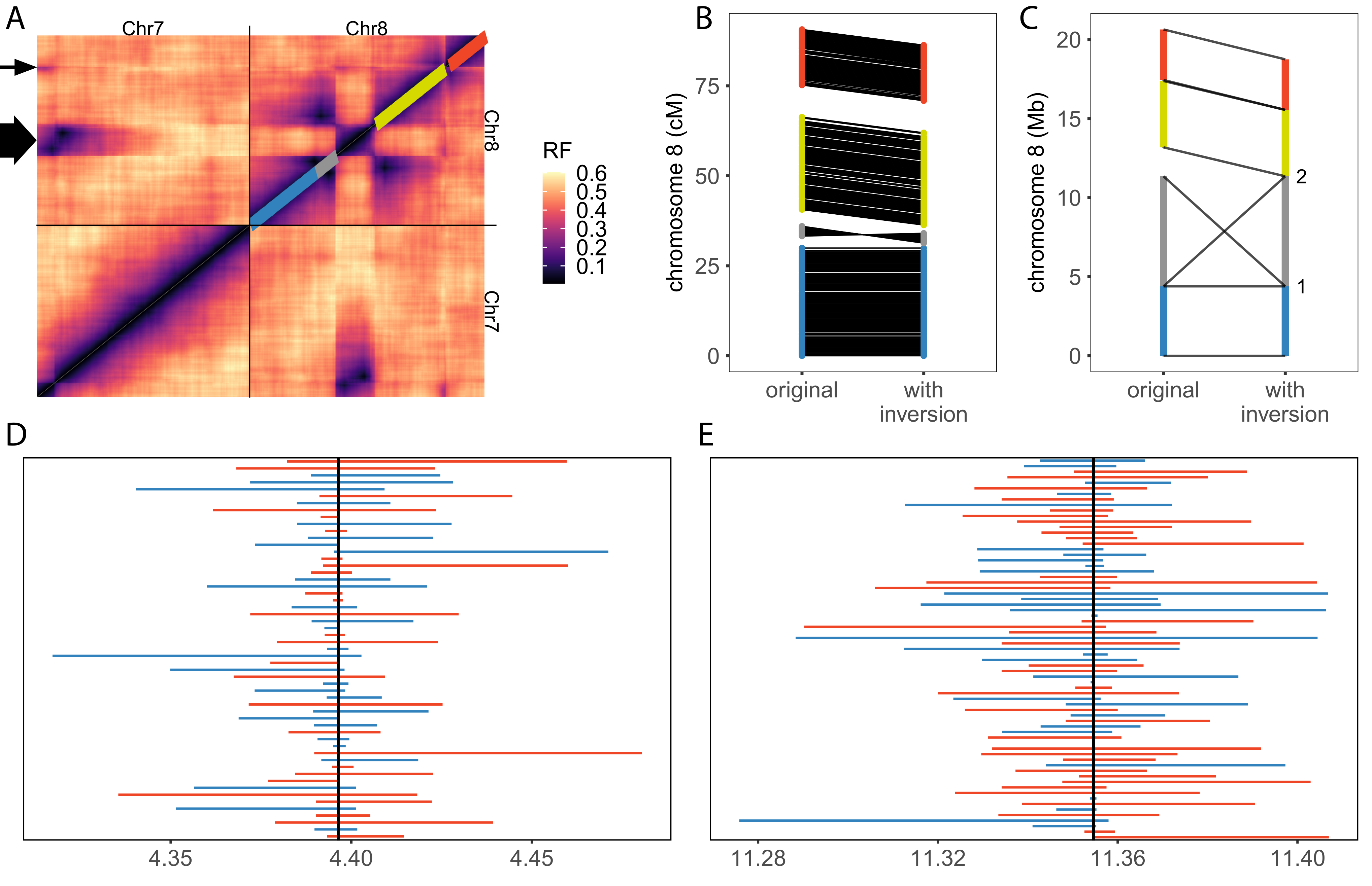

Supplement: S6 Fig — (A) The RF of genetic markers within the Ox × Az1 RIL population reveal 2 regions on chromosome 8 that most likely belong to chromosome 7 instead of previous assignment to chromosome 8 (arrows). In addition, markers of the pericentromeric region (gray diagonal line) might be inverted. (B, C) Genetic (B) and physical (C) maps based on previous (left side) and new assembly (right side) of chromosome 8. Regions colored in red, yellow, and blue depict unchanged segments of the map, whereas the region in gray color corresponds to the inverted segment. A genetic map with inverted order of markers shows reduced genetic length supporting the inverted assembly (B). The breakpoints of the predicted inversion indicated by 1 and 2 have been previously uncovered by consistent breakpoints in Nanopore long reads mapped to the original assembly of chromosome 8 (C). (D, E) Images representing Nanopore reads (positive strand–blue; negative strand -red) that span the regions 1 (D) and 2 (E) of the reassembled chromosome 8 and confirm the correctness of the new assembly. The data underlying the graphs shown in the figure can be found at https://doi.org/10.5281/zenodo.7907435. Az1, Azores1; Ox, Oxford; RF, recombination fraction; RIL, Recombinant Inbred Line. (TIFF) [file pbio.3002191.s006.tiff]
